# Shifting Dietary Ecologies of Early Ungulates Across the Paleocene–Eocene Thermal Maximum in the Bighorn Basin, Wyoming

**DOI:** 10.1002/ece3.74613

**Published:** 2026-11-03

**Authors:** Andrew Schwartz, Larisa R. G. DeSantis, Rob S. Scott

**Affiliations:** ^1^ Department of Anthropology Rutgers, The State University of New Jersey–New Brunswick New Brunswick New Jersey USA; ^2^ Department of Biological Sciences Vanderbilt University Nashville Tennessee USA; ^3^ Department of Earth and Environmental Sciences Vanderbilt University Nashville Tennessee USA

**Keywords:** climate change, dietary ecology, Paleocene–Eocene Thermal Maximum, Perissodactyla, ungulates

## Abstract

Understanding the interplay between climate change and ecology is crucial when attempting to reconstruct the evolutionary history of species, as well as predicting how modern species may fare in light of current anthropogenic climate change and habitat destruction. Here we use dental microwear texture analysis (DMTA) to infer the foraging ecologies of three lineages of early ungulates (the phenacodontids *Copecion* and *Ectocion* and the early perissodactyl *Sifrhippus*) across the Paleocene–Eocene Thermal Maximum (PETM) in the Bighorn Basin of western North America. The PETM was a period of rapid warming and aridification 56 million years ago that resulted in considerable evolutionary changes within these lineages, including phylogenetic dwarfing. Our results interpret both lineages of phenacodontid as frugivorous “mixed feeders” that likely incorporated a large diversity of plant species and tissues, while *Sifrhippus* shows strong evidence of frugivory. This is further supported by greater complexity and lesser scale of maximum complexity in both species of *Sifrhippus* compared to the co‐occurring species of phenacodontids. Notably, only minor changes in dental microwear across the PETM were recovered, limited to textural fill volume in *Ectocion*. These data show that dietary ecology was mostly unchanged within these lineages during the PETM, despite considerable floral turnover and competition from immigrant species. The results of our study detail how herbivorous mammals adapt to intensive climatic and environmental disruptions, hinting at possible responses of modern fauna to present and future anthropogenic climatic and environmental breakdown.

## Graphical abstract

Understanding the interplay between climate change and ecology is crucial when attempting to reconstruct the evolutionary history of species. Here we use dental microwear texture analysis to infer the foraging ecologies of three lineages of early ungulates across the Paleocene–Eocene Thermal Maximum. We recovered differences in foraging guilds and only minor changes in dental microwear across the PETM, reconstructing evidence of ecological niche partitioning and differing dietary responses to intensive climatic and environmental disruptions.

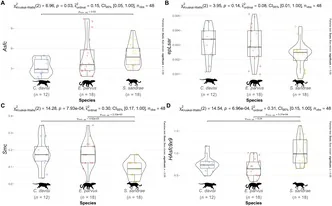

## 1 Introduction

Anthropogenic effects on terrestrial ecosystems have been catastrophic, exemplified through climate change, habitat destruction, and the introduction of non‐native species. The consequences have been dire, with many species becoming at risk or succumbing to extinction (Cooke et al. 2017; Kerr and Currie 1995; Matthews et al. 2024). Therefore, it is crucial that we understand how fauna have responded to these environmental disruptions in the past in order to predict patterns of adaptation in the future to better focus our conservation efforts.

The Paleocene–Eocene Thermal Maximum (PETM) serves as an informative model for the effects of rapid global warming on the biology, ecology, and evolution of mammals over a geologically fine time scale. Roughly 56 million years ago, there was a large carbon isotope excursion (CIE) resulting from a massive release of isotopically light carbon. This caused average global surface temperatures to rise 5°C–8°C over an onset period of just a few thousand years, with these conditions persisting over a 100,000 year “body” and a ~150,000 years “recovery” interval (Li et al. 2022; Piedrahita et al. 2025; Tierney et al. 2022; Zeebe and Lourens 2019). In western North America where records of the event are quite complete, precipitation plummeted with evidence of progressive aridification through the PETM, peaking during the latter half of the event (Kraus et al. 2013; Kraus and Riggins 2007; Xu et al. 2025). There is also evidence for increased frequency of extreme seasonal precipitation events across the PETM (Carmichael et al. 2018; Schmitz and Pujalte 2007). As these conditions mirror the modern climate crisis, the PETM serves as a relevant case study to explore how climatic breakdown affects the biology, ecology, and evolution of modern ecosystems.

In the Bighorn Basin of Wyoming, there is an abundance of localities preserving fossil plants and animals from the late Paleocene (Clarkforkian) – early Eocene (Wasatchian) North American Land Mammal ages (NALMA), in addition to a relatively complete stratigraphic column through the PETM (Bown et al. 1994). Systematic collection over the past century has resulted in the recovery of thousands of mammal fossils from the early Cenozoic. This makes the region an excellent testing ground for tracing both species and ecosystem evolution in response to climate change (Vitek et al. 2021; Whittingham et al. 2024), as has been done previously for taxa across the PETM (Schwartz et al. 2025).

There was considerable floral change in the Bighorn Basin during the PETM. During the late Paleocene, the climate was wet and subtropical, with abundant deciduous and evergreen broadleaf forests dominated by temperate‐region families such as Betulaceae, Cupressaceae, Hamamelidaceae, and Platanaceae (Korasidis and Wing 2023; Wing and Currano 2013). The PETM coincided with considerable local and regional extirpation of mesophytic plants (particularly conifers), with floral turnover estimated at over 80% (Korasidis and Wing 2023; Wilf 2000; Wing and Currano 2013). During the onset of the CIE, floral composition suggests a seasonally dry tropical climate with open deciduous forests and savannas abundant with ferns and *Celtis* trees (Korasidis and Wing 2023). During the “body” of the PETM, further immigration took place in the form of thermophilic and dry‐tolerant tropical species from the families Arecaceae, Fabaceae, and Hernandiaceae (Korasidis et al. 2025). The recovery interval and post‐PETM stage of the early Eocene represented a return to a wet subtropical climate composed of a mixed floral community of tropical and temperate lineages occupying an open canopy (Korasidis and Wing 2023; Secord et al. 2008). In many ways, this community possessed similarities to the pre‐CIE Paleocene, but species accumulations show the lowest richness during the early Eocene compared to both the latest Paleocene and PETM (Korasidis and Wing 2023).

Faunal communities also underwent considerable alteration during the PETM. In the latest Paleocene Clarkforkian NALMA, the Bighorn Basin hosted a diverse composition of species from archaic and modern mammalian orders (Rose 1981). Ubiquitous to the Paleocene was Phenacodontidae: a family of archaic ungulates traditionally classified within the polyphyletic “Condylarthra” but now often recovered as close relatives of Perissodactyla due to shared dental characteristics, though the latter exhibits morphology suggesting advanced shearing adaptation including greater lophodonty and shearing crest development (Bai et al. 2019; Halliday et al. 2017; Radinsky 1966). They were contemporaneous with other archaic ungulates including Hyopsodontidae, Mesonychia, and Pantodonta, as well as stem‐Carnivora and the euarchontan Plesiadapiformes (Gingerich 1989; Rose 1981).

Faunal changes corresponding to the PETM in the Bighorn Basin were threefold: extinction, immigration, and phylogenetic dwarfing. Extinctions among native taxa were relatively minimal, mainly restricted to the crocodile‐like *Champsosaurus* and Plesiadapidae (Gingerich 2003). This period is notable for the first appearances of Artiodactyla, Perissodactyla, and Primates in the fossil record, occurring simultaneously across the Holarctic (Bowen 2002; Smith et al. 2006). Relatively few terrestrial extinctions among native species despite this invasion suggest niche unsaturation within the Bighorn Basin, possibly due to abiotic perturbations that reduced population levels, increased niche conservatism, and niche contraction (Fraser and Lyons 2020).

A particularly notable effect of the PETM was dwarfing across numerous lineages of mammals in the Bighorn Basin. Over a dozen native and immigrant lineages experienced body mass reduction during the PETM (some as much as 80%), followed by body mass increase during the PETM recovery (Gingerich 2003; Secord et al. 2012; Solé et al. 2021). The ultimate causes of this dwarfing are debated between Bergmann's Rule, reduced primary productivity through increased aridity or plant physiological changes, and northward immigration/displacement of natives by closely related smaller taxa. Proponents of Bergmann's Rule argue that the increased temperatures in the Bighorn Basin during the PETM drove body mass reduction as an adaptation for better heat dissipation (Bergmann 1847; Secord et al. 2012). Indeed, trends of body mass changes in the early perissodactyl *Sifrhippus sandrae* are closely correlated with ambient air temperature proxies (Secord et al. 2012). However, size differences distinguishing some PETM taxa from their early Wasatchian counterparts would require temperature increases nearly three times that which is reconstructed for the PETM (Gingerich 2003; Koch 1986).

Those that favor an explanation involving changes in precipitation argue that increased aridity during the PETM both directly and indirectly created selection pressure for smaller body sizes. Positive correlations between precipitation and body size have been observed in multiple mammalian orders, likely reflecting reduced primary productivity (Alhajeri and Steppan 2016; Roycroft et al. 2020; Yom‐Tov and Geffen 2006). This “resource‐rule” suggests that larger body size is a consequence of greater resource availability (food and water), with the inverse reflecting lesser resource availability (Alston et al. 2023; Huston and Wolverton 2011; Mcnab 2010). Models simulating floral adaptational responses support changes in resource availability through adaptational lags and reductions in primary productivity during the PETM “body” in the Bighorn Basin (Rogger et al. 2025).

Additionally, increased temperatures and partial pressures of carbon dioxide during the PETM likely affected plant biology. Plants such as legumes (Fabaceae) grown under increased temperatures were found to produce decreased seed yields despite elevated levels of carbon dioxide, primarily due to fertility issues and reduced seed size/filling (Prasad et al. 2002; Sita et al. 2017; Vara Prasad et al. 2003). Drought has been shown to amplify these effects of heat stress (Hatfield and Prueger 2015; Suzuki et al. 2014). Additionally, abrupt changes in extreme seasonal precipitation events brought about by the PETM may have negatively impacted seed production, making food availability more unpredictable (Hanley et al. 2019; Schmitz and Pujalte 2007; Tierney et al. 2022).

Plants grown under elevated partial pressure carbon dioxide conditions show reduced protein content when grown under similar conditions to the PETM (Royer 2001; Stiling and Cornelissen 2007). Cellulose‐rich, protein‐poor vegetation is more difficult to digest and may result in slower growth and reproduction rates among herbivores, selecting for a reduced mature body size (Owensby et al. 1996). Therefore, reduced primary production coupled with increased aridity would have selected for smaller body sizes due to a reduction in resource availability.

However, these explanations are also not well supported. There is little correlation between increases in aridity or partial pressure of carbon dioxide and the body mass of *Sifrhippus* across the PETM, suggesting that resource availability was likely not the main driver of body mass change in this lineage (Cui et al. 2011; Secord et al. 2012). Additionally, the Jarman/Bell principle suggests that an increase (rather than decrease) in size is the predicted response to decreasing plant nutrition as greater quantities of low‐quality vegetation are needed to meet an animal's nutritional requirements (Gaulin 1979). Finer‐scale examination of other taxa within the PETM is needed to clarify the influence of aridity and carbon dioxide partial pressure on body mass.

Finally, some argue that the climatic conditions of the PETM caused widespread northward migration of taxa, with smaller species replacing their more northern counterparts. In this scenario, the appearance of “dwarf” taxa in the Bighorn Basin represents biogeographic range shifting rather than a signal of directional selection during the PETM. Though support for this hypothesis has come from the apparent presence of the otherwise PETM‐specific taxon *Ectocion parvus* in the Clarkforkian Piceance Creek Basin of Colorado (Burger 2012), the species designation of this material is controversial. Though these specimens compare well to *E. parvus* from the PETM of the Bighorn Basin, they also show considerable similarity to the diminutive *Ectocion stockeyae* from the late Tiffanian of Alberta, Canada (Montellano‐Ballesteros et al. 2021). Because overlapping material is not represented in the Piceance Creek Basin specimens, we cannot eliminate the possibility that these specimens represent a southern population of *E. stockeyae* (Burger, personal communication). Notably, no other PETM‐specific taxa have been recovered from southern Paleocene‐aged exposures while a specimen of *Ectocion* intermediate in size between *E. parvus* and *E. osbornianus* was recovered from the PETM recovery interval in the Bighorn Basin, hinting at a return to pre‐PETM body mass in this lineage (Van Der Meulen et al. 2020).

Ungulates from the late Paleocene–early Eocene in the Bighorn Basin are ideal candidates to test these hypotheses related to the biological effects of the PETM. The phenacodontids *Copecion* and *Ectocion* both persist across the Paleocene–early Eocene and are abundant during the PETM (Gingerich 2003; Rose 1981). The Clarkforkian *Copecion brachypternus* and *Ectocion osbornianus* are replaced by the diminutive *Copecion davisi* and *Ectocion parvus* (respectively) in the Bighorn Basin during the PETM (Gingerich 1989). During the following recovery period, the larger *C. brachypternus* and *E. osbornianus* reappear in the Bighorn Basin, while these PETM‐specific taxa disappear (Gingerich 2003).

The taxonomic status of phenacodontids otherwise restricted to the PETM is the subject of debate due to size being their main diagnostic trait (Gingerich 1989). Assuming phenacodontids in the Bighorn Basin form genuine ancestor–descendant lineages across the PETM (Gingerich 2003; Wood et al. 2007), taxa need to either be subsumed into single species that vary morphologically over time (*C. brachypternus* and *E. osbornianus*), or unique taxa need to be assigned to each biozone. For example, the National Museum of Natural History (Washington D.C., USA) has attempted to remedy this by assigning Clarkforkian *Ectocion* to the species *Ectocion ralstonensis*, PETM specimens to *E. parvus* , and post‐PETM Wasatchian specimens to the type species *E. osbornianus* (Wing, personal communication). As resolving taxonomic uncertainties is beyond the scope of this study, we make no recommendations as to specific designation. Instead, we specify the taxon's temporal age through inclusion of NALMA via subscript (*C. brachypternus*_Cf_, *E. osbornianus*_Wa_, etc.).

The early perissodactyl *Sifrhippus* is also an ideal taxon to study the effects of the PETM on biology and ecology. Unlike phenacodontids, this taxon abruptly appears in the Bighorn Basin during the PETM, likely immigrating from Eurasia across high‐latitude dispersal routes opened by warming near the onset of this event (Bowen 2002; Koch et al. 1992). Additionally, fossils of *S. sandrae* have been collected with high‐resolution stratigraphy in the Bighorn Basin, enabling the tracing of body mass reduction through the body of the PETM, followed by body mass increase during the recovery period through the plausible anagenetic descendent *Sifrhippus grangeri* (Secord et al. 2012). Most recently, Tissier and Smith (2026) synonymized numerous genera of early Eocene “equids” (*Arenahippus*, *Minippus*, and *Sifrhippus*) into the genus *Pliolophus* based on four unambiguous synapomorphies, whilst maintaining distinctions between species. They also pulled this clade out of Equoidea entirely, instead recovering *Pliolophus* in a basal position within Perissodactyla (Tissier and Smith 2026). While this is an intriguing development toward disentangling the phylogeny of early Cenozoic Perissodactyla, this does not affect our analysis as a close relationship between former species of *Sifrhippus* was still supported. Ergo we continue use of “Sifrhippus” in reference to the PETM/early Eocene lineage of North American perissodactyls traditionally classified as *Hyracotherium sandrae* and *Hyracotherium grangeri* until further confirmation of the validity of these genera synonymizations.

All three ungulates share general dental and postcranial similarities in morphology, likely reflecting similar niches in late Paleocene–early Eocene environments. Compared to the bunodont, bulbous cusped dentition of *Copecion*, *Ectocion* exhibited greater lophodonty, possessing a W‐shaped ectoloph as well as a deeper trigonid basin (Radinsky 1966). While these morphologies suggest frugivory in both taxa, differences hint at greater browsing and shearing of tough‐plant material in *Ectocion* (Rose et al. 2012). Though the dentition of *Sifrhippus* is also brachy‐bunodont, it differs in possessing connective ridges between cups, a condition termed “bunolophodonty” (Semprebon et al. 2016). This combination of traits suggests that early perissodactyls like *Sifrhippus* were mixed frugivore‐browsers, supported by previous studies of dental microwear via scanning electron microscopy (Semprebon et al. 2016; Solounias and Semprebon 2002). While the lophodonty of *Ectocion* indicates greater specialization for vertical shear, the highly developed crest and jaw musculature suggest greater importance on transverse shear for early perissodactyls (Radinsky 1966).

The coexistence of three morphologically similar herbivorous lineages in the Bighorn Basin raises questions regarding how these taxa partitioned dietary resources, as the competitive exclusion principle predicts that species occupying similar ecological niches cannot coexist indefinitely at comparable population levels (Gause 1934; Hardin 1960). Although *Copecion*, *Ectocion*, and *Sifrhippus* were all relatively abundant during the PETM despite substantial climatic and floral changes (Gingerich 2003; Rose 1981; Secord et al. 2012), the ecological mechanisms that permitted their coexistence remain poorly understood. Following the PETM, all three lineages increased in body mass; yet phenacodontids ultimately declined and disappeared during the Wasatchian while perissodactyls persisted (Chew 2009, 2015; Schankler 1981). Whether differences in dietary ecology or digestive efficiency contributed to these contrasting population trajectories remains unknown.

To investigate the effects of the PETM on the ecologies of mammals, we conducted dental microwear texture analysis (DMTA) on specimens of early ungulates from the late Paleocene–early Eocene in the Bighorn Basin. DMTA has been effectively used to reconstruct dietary ecology and change over time in modern (Scott 2012; Scott et al. 2005), historic (Burtt and Desantis 2022; Schwartz et al. 2024), and prehistoric (Desantis et al. 2019; Schwartz et al. 2025) records. DMTA records perimortem feeding events and is overwritten after roughly 2–3 weeks via the “last supper effect” (Winkler, Schulz‐Kornas, et al. 2020), likely reflecting diet shortly prior to death rather than a compilation of lifelong dietary patterns.

We expect dental microwear from these early ungulates to closely resemble data from extant frugivores and browsers. While the post‐canine dentition of *Copecion* is bunodont and lacks substantial shearing crest development, both *Ectocion* and *Sifrhippus* exhibit minor to moderate lophodonty (Gingerich 1989; Semprebon et al. 2016; Thewissen 1990). Compared to the relatively flat, low‐cusped bunodont teeth that involve complex crushing and grinding, incipient lophodonty enhances the shearing of tough foods such as leaves as they wear down (Ungar 2010). Furthermore, similarities to modern grazers would not be expected due to the open‐forested paleoenvironment of the Bighorn Basin across the Paleocene–Eocene where monocotyledonous plants were rare or otherwise minor floral components (Wing and Currano 2013; Wing et al. 2005).

An additional challenge in applying modern dental microwear reference datasets to ancient ecosystems is that modern plant communities often differ substantially from those present in the deep past, including the Paleocene–Eocene of North America. Early Cenozoic ecosystems contained abundant plant groups that are uncommon or ecologically marginal in modern terrestrial herbivore communities such as ferns (Polypodiidae) and horsetails (Equisetidae) within the Bighorn Basin (Korasidis and Wing 2023; Wing and Currano 2013). In modern ecosystems, these plants are rarely consumed by mammals, creating considerable uncertainty regarding how their mastication influences dental microwear formation. Importantly, the material properties of these plants suggest that they could produce microwear textures overlapping with tough dicotyledonous plants represented in modern DMTA reference datasets. Fern leaves have relatively high bulk elastic moduli, indicating substantial rigidity, while horsetail stems have bending elastic moduli substantially higher than those reported for grasses, ferns, and woody plants (Daegling et al. 2016; Nadal et al. 2018; Spatz et al. 1998). Because experimental studies directly examining dental microwear formation during fern or horsetail mastication are currently lacking, the extent to which microwear produced by these plants overlaps with or differs from patterns represented in modern DMTA reference datasets remains unknown.

We also predict dental microwear data to suggest greater durophagy during the PETM within all three lineages. Due to reductions in primary production and migration of flora into/out of the Bighorn Basin, it may be expected that herbivores would shift toward more generalized and opportunistic foraging to meet their daily caloric needs (Codron et al. 2007; Kartzinel and Pringle 2020). In light of the hotter, more arid conditions of the PETM, these herbivores may have turned to fallback foods: non‐preferred resources of varying quality that are exploited during periods of food scarcity (Marshall et al. 2009). These may be resources that persist longer or in greater abundance in more arid conditions such as bark, seeds, or nuts, all of which create pitted, complex dental microwear indicative of durophagy (Scott et al. 2006; Winkler et al. 2022).

Elucidating changes in dietary ecologies over time for these early ungulate lineages is invaluable toward understanding the effects of emerging niche competition as well as rapid climatic warming, aridity, and habitat loss on mammalian biology and ecology. By reconstructing foraging ecology, we can infer niche overlap between taxa and how they may have mitigated competition. Recovering changes in diet across the PETM is crucial toward understanding how animals adapt to climatic and environmental disruptions that are similar to those experienced in the 21st century.

## 2 Methods

### 2.1 Sample Preparation

Specimens sampled for this study were housed at Denver Museum of Nature and Science (DMNH), University of Florida Museum of Natural History (UF), and University of Michigan Museum of Paleontology (UMMP). Refer to dataset available on Dryad (https://datadryad.org/share/LINK_NOT_FOR_PUBLICATION/JZpowabG_6UyXa_Ap0mCZR0‐am7EFXkN4IOltziYIa4) for specimen numbers and institutional affiliation. Although fossils derive from multiple locations across the Bighorn Basin, these localities likely represent broadly similar floodplain and backswamp paleoenvironments (Wang et al. 2022), which shared similar floral compositions (Korasidis and Wing 2023; Wing and Currano 2013). Previous research has shown that dental microwear in herbivorous ungulates accumulates in a similar fashion across homologous facets regardless of molar position or placement in the maxilla or mandible (Kelly et al. 2021; Merceron et al. 2004). Therefore, we included both upper and lower first and second molars in our analysis. Specimens with well‐developed phase‐II wear facets were selected (Figure 1), with special attention paid to facets 5 (buccal hypocone; lingual protoconid) and 10 (buccal protocone; lingual hypoconid) in accordance with previous studies (Butler 1952; Hunter and Fortelius 1994; Kelly et al. 2021). In total, we sampled 4 *C. brachypternus*_Cf_, 12 *C. davisi*_PETM_, 14 *C. brachypternus*_Wa_, 15 *E. osbornianus*_Cf_, 18 *E. parvus* _PETM_, 14 *E. osbornianus*_Wa_, 18 *S. sandrae*_PETM_, and 11 *S. grangeri*_Wa_. The majority of phenacodontid specimens utilized in this study derive from Gingerich (2003), with notable underrepresentation of *C. brachypternus* from the Clarkforkian NALMA. This taxon seems to be considerably less common than contemporaneous *E. osbornianus*, indicating that further collection of *C. brachypternus* from the Clarkforkian is crucial to fully evaluate ecological change within this lineage across the PETM in the Bighorn Basin.

**FIGURE 1:**
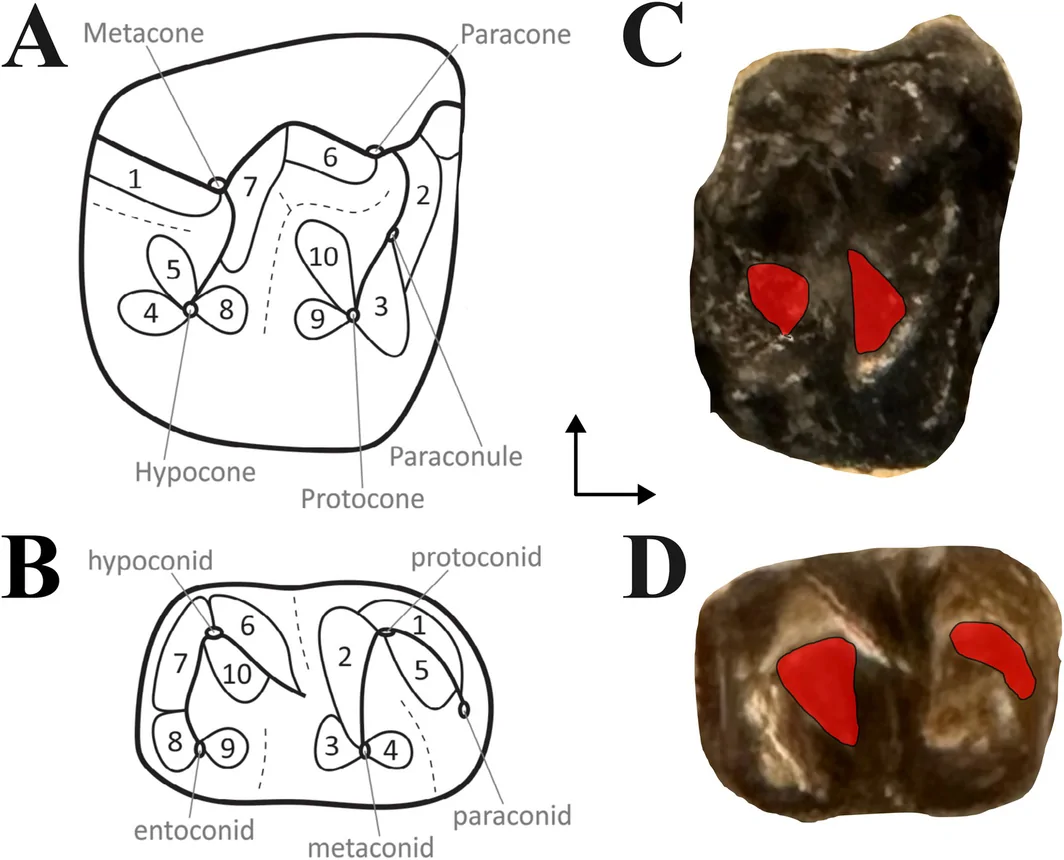
(A, B) Schematic illustration of the wear facets on the upper and lower first molar surfaces of a stem perissodactyl adapted from Butler (1952) and Schultz et al. (2018). (C, D) Upper and lower molars of *Copecion brachypternus* with targeted wear facets (5, 10) highlighted in red. Arrows indicate mesial (right), buccal (top).

Careful consideration was taken to exclude fossil specimens that exhibited signs of taphonomic processes such as exposure to acid, trampling, sediment abrasion, and fossil laboratory preparation as all of these are capable of altering dental microwear features through polishing, attrition, breakage, and etching (King et al. 1999; Weber et al. 2021, 2022). However, many of these taphonomic processes are easily identifiable through diagnostic features (cracks, varnish residue, drill marks, irregular striations) and by the fact that alteration will not be limited to the occlusal surface of the tooth (Micó et al. 2024; Teaford 1988; Weber et al. 2021). Specimens in which considerable taphonomic alteration on dental wear facets occurred were identified and removed from our sample.

Across the PETM in the Bighorn Basin, the earliest Wasatchian is typically divided into three biozones: Wa‐M, Wa‐0, and Wa‐R. Wa‐M is the earliest and shortest biozone and is characterized by the presence of the phenacodontid *Meniscotherium priscum* (Gingerich and Smith 2006). Wa‐0 is the middle PETM biozone and is notable for the first appearance of several dwarf taxa within the Bighorn Basin (Gingerich 1989). Wa‐R encompasses the highest stratigraphic levels of the PETM and contains a fauna intermediate between those of Wa‐0 and Wa‐1 (Van Der Meulen et al. 2020). All specimens analyzed here that are assigned to the PETM derive from the “main body,” corresponding to the Wa‐0 biozone. To distinguish these specimens from those assigned to the remainder of the Wasatchian NALMA, we refer to individuals from Wa‐0 as PETM specimens throughout this study.

Sample preparation protocol is outlined by (Desantis et al. 2012). Teeth were first cleaned with a toothbrush soaked with 70% isopropyl alcohol. Acetone was then applied using a small cotton swab to remove preservatives such as Paraloid B‐72. Surface molds were prepared using President's Jet regular body polyvinylsiloxane dental impression material (Coltène‐Whaledent Corp., Cuyahoga Falls, OH, USA). High‐resolution translucent casts were poured using Epo‐tek 301 epoxy resin and hardener (Epoxy Technologies Corp., Billerica, MA, USA).

### 2.2 Dental Microwear Texture Analysis (DMTA)

Wear facets on these high‐resolution casts were scanned for dental microwear using a Sensofar PLμ neox confocal profiler (Solarius Inc., Sunnyvale, CA, USA) at Vanderbilt University. Specimens were scanned in three dimensions across nine areas and stitched together, leveled, and subdivided into four adjacent areas of equal size (102 × 138 μm^2^) for a total sampled area of 204 × 276 μm^2^ in accordance with protocol from previous studies (Scott et al. 2006), an area that fits well within the wear facets of these archaic ungulate teeth. These scans output four files (.sur) available for DMTA using Sfrax and Toothfrax software. By combining three‐dimensional scans of wear facet surfaces with scale‐sensitive fractal analysis, DMTA is able to analyze the surface texture of these high‐resolution models (Scott et al. 2005; Ungar et al. 2003), while also being superior to two‐dimensional analyzes at discriminating frugivores and intermediate feeders from grazers and browsers (Desantis et al. 2013). Three‐dimensional microwear is visualized through C3D images (Figure 2).

**FIGURE 2:**
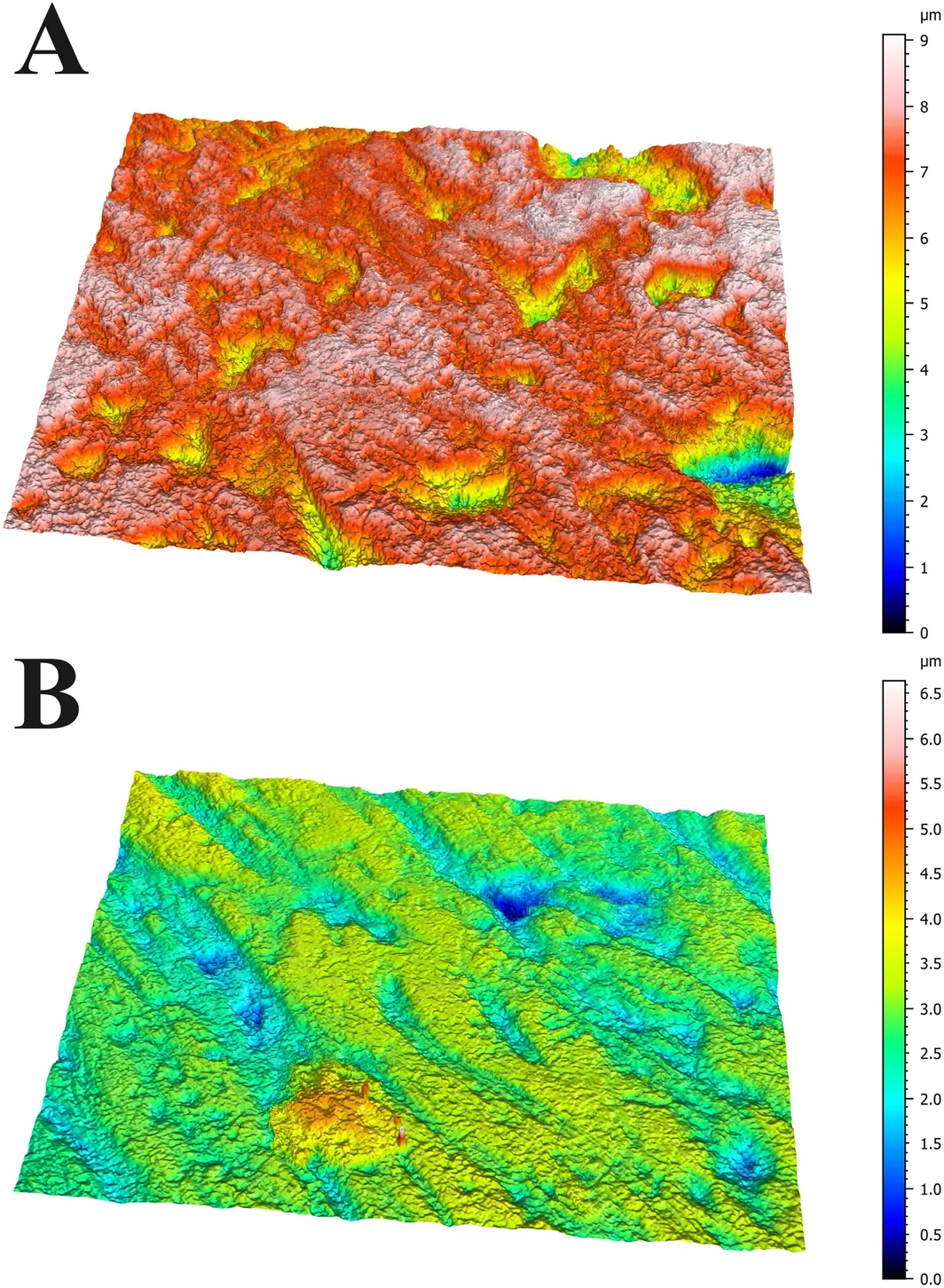
3D digital elevation dental microwear surface models of *Sifrhippus* exhibiting high (A) complexity and (B) anisotropy. Both models represent 204 × 274 μm in area with depth scales noted to the right of each image.

We utilized five DMTA variables in our analysis: *Asfc*, *epLsar*, *Smc*, *Tfv*, and *HAsfc*_9×9_. See Scott et al. (2006) for more detailed descriptions of these variables. Complexity (*Asfc*) measures the maximum change in surface roughness as the scale of observation increases, essentially quantifying the number of features (pits and scratches) on a wear surface. Complexity tends to be highest in frugivores and browsers with hard/brittle diets of seeds, nuts, woody materials, and hard fruits. Anisotropy (*epLsar*) quantifies the degree to which features share a similar orientation (i.e., parallel scratches) and tends to be highest in grazing herbivores with tough diets consisting of grass or very tough leaves. Scale of maximum complexity (*Smc*) measures the range of scales in which *Asfc* is calculated and is greater in surfaces dominated by large features combined with fewer small features, which tends to be highest in browsers and grazers and lowest in frugivores and generalists (Scott et al. 2009). Textural fill volume (*Tfv*) quantifies the volume of wear by measuring the number of large (10 μm diameter) and small (2 μm diameter) square cuboids needed to fill these features. Surfaces with high *Tfv* likely have larger and deeper features, often highest in browsers and frugivores. Heterogeneity of complexity (*HAsfc*) quantifies the amount of variation in wear features across adjoining surfaces and is higher in surfaces with a large variety of microwear across the scan, particularly seen in browsers. We use a scale of 9 cells by 9 cells (*HAsfc*_9×9_), representing heterogeneity over a surface of 81 cells.

We compared DMTA values of Paleocene–Eocene ungulates to a dataset of African bovids previously published in Scott et al. (2012), modified with additional specimens in Schwartz et al. (in press) to reconstruct diet of these extinct taxa. These data consisted of 585 specimens from 25 species of extant African bovid, divided into six groups based on observed foraging ecology (Scott 2012, and references therein). Bovids and equids have both evolved to chew via a primarily single‐phase transverse shearing motion and have similar, flat occlusal planes on their molars, suggesting wear signals may be comparable if not perfectly analogous, and dental microwear comparisons should mostly reflect a dietary rather than phylogenetic signal (Kelly et al. 2021; Mihlbachler et al. 2016). That being said, phenacodontids exhibited more primitive mastication patterns consisting mostly of compression rather than translation (Rensberger 1986), which may introduce uncertainty when compared to bovids.

It is important to note that bovids differ from non‐ruminants as they continuously regurgitate boluses of semi‐digested food for further mastication (“chewing cud”). Mihlbachler et al. (2016) contend that this process may result in a stronger relationship between microwear and diet due to the removal of inorganic particles in the reticulorumen, leading to wear patterns more strictly related to the abrasive properties of food (Kubo et al. 2025; Valerio et al. 2022). Additionally, equids exhibit diversity in masticatory cycles based on diet, introducing further uncertainty into dental microwear comparisons between bovids and perissodactyls (Bonin et al. 2007). Nevertheless, Scott et al. (2012) is the most comprehensive comparative dataset of dental microwear across extant ungulate foraging guilds and should be utilized to evaluate potential diets in extinct herbivorous ungulates when alternative comparative datasets are not available.

In modern African ecosystems, bovid foraging guilds are based primarily on the prevalence of monocots, dicots, and fruit consumed (Gagnon and Chew 2000). Obligate and variable grazers predominately consume monocots (primarily grasses), while browsers consume more than 70% dicots (herbaceous vegetation). Frugivores consume more than 70% fruit (with minor dicot incorporation). Browser‐grazer intermediates consume roughly 50% monocots, while generalists' diets are composed of at least 20% monocot, dicot, and fruit each. We do note that modern African ecosystems are not perfect approximations for early Cenozoic environments in the Bighorn Basin due to the particular rarity of grasses in the latter (Crepet and Feldman 1991). Therefore, we are unlikely to recover close affinities of extinct ungulate diets to modern variable and obligate grazers.

### 2.3 Statistical Analysis

We compared DMTA between and across taxa to infer dietary niche as well as trace changes across the PETM. DMTA data were not normally distributed (Shapiro–Wilk tests, Table 1). Thus, nonparametric tests (e.g., Kruskal–Wallis) were used to compare differences between taxon, guild, and NALMA. When significant differences were recovered, Dunn's procedure was used to conduct multiple comparisons (Dunn 1964). We employed a Benjamini–Hochberg procedure (with false discovery rate set at 0.05) to address increased Type I errors (Benjamini and Hochberg 1995). This procedure is preferable to approaches that seek to control the family‐wise error rate due to being less conservative and producing fewer Type II errors (Williams et al. 1999) and has been increasingly utilized in studies of DMTA (Adams et al. 2020; Schwartz et al. 2025; Winkler et al. 2022).

**TABLE 1:** Descriptive statistics.

Taxon – NALMA	Statistic	*n*	*Asfc*	*epLsar*	*Smc*	*Tfv*	*HAsfc*_9×9_
*C. brachypternus*_Cf_	Median	4	4.10	0.0027	0.1309	9728	0.859
	Mean		3.79	0.0036	0.1584	11,070	0.917
	SD		0.842	0.0022	0.0851	5963	0.364
	Skewness_Fisher_		−0.99	1.01	0.9549	0.726	0.346
	p for normality (Shapiro–Wilk)		0.138	0.066	0.186	0.378	0.587
*C. davisi*_PETM_	Median	12	2.89	0.0034	0.1722	7561	0.734
	Mean		3.19	0.0032	0.1791	8544	0.759
	SD		1.19	0.0011	0.0906	3315	0.182
	Skewness_Fisher_		0.12	−0.386	0.1991	0.147	0.796
	p for normality (Shapiro–Wilk)		0.368	0.653	0.5753	0.252	0.512
*C. brachypternus*_Wa_	Median	14	3.38	0.0029	0.1963	8843	0.642
	Mean		4.06	0.0028	0.1816	7687	0.740
	SD		2.60	0.0017	0.1136	5434	0.319
	Skewness_Fisher_		0.349	1.002	1.3274	0.187	1.506
	p for normality (Shapiro–Wilk)		0.182	0.0930	**0.0055**	0.2067	**0.0233**
*E. osbornianus*_Cf_	Median	15	2.63	0.0027	0.1722	4457	0.738
	Mean		3.26	0.0031	0.1837	4668	0.888
	SD		1.64	0.0012	0.0813	3804	0.365
	Skewness_Fisher_		1.88	0.8616	−0.2089	0.816	0.753
	p for normality (Shapiro–Wilk)		**0.0015**	0.1179	0.1175	0.215	**0.0363**
*E. parvus*_PETM_	Median	18	3.58	0.0029	0.1722	10,277	0.648
	Mean		4.70	0.0030	0.1761	10,139	0.664
	SD		2.78	0.0013	0.0766	4127	0.203
	Skewness_Fisher_		1.007	0.0603	0.1360	−0.457	0.322
	p for normality (Shapiro–Wilk)		**0.0055**	0.6608	0.6316	0.9329	0.376
*E. osbornianus*_Wa_	Median	14	3.09	0.0030	0.2204	11,541	0.575
	Mean		4.12	0.0032	0.3400	10,758	0.873
	SD		2.75	0.0015	0.2489	5758	0.625
	Skewness_Fisher_		0.88	0.4128	1.1926	−0.535	1.980
	p for normality (Shapiro–Wilk)		**0.0094**	0.616	**0.0047**	0.1338	**0.0004**
*S. sandrae*_PETM_	Median	18	4.71	0.00249	0.0896	9048	0.986
	Mean		4.98	0.00235	0.0919	8456	1.04
	SD		1.75	0.00090	0.0494	5692	0.307
	Skewness_Fisher_		0.547	0.07600	−0.1702	−0.117	0.321
	p for normality (Shapiro–Wilk)		0.316	0.450	0.1064	0.224	0.125
*S. grangeri*_Wa_	Median	11	4.99	0.00225	0.1240	8463	0.786
	Mean		5.76	0.00281	0.1259	6753	0.805
	SD		2.90	0.00143	0.0419	6161	0.312
	Skewness_Fisher_		1.245	0.9675	−0.1425	0.376	0.688
	p for normality (Shapiro–Wilk)		0.096	0.160	0.0741	0.146	0.448

*Note:* Bold indicates *p* < 0.05.

To evaluate whether non‐significant results could be attributable to limited sample sizes, we conducted simulation‐based power analyzes. Power was estimated using 5000 bootstrap simulations while maintaining the observed group‐size ratios for each taxon per biozone. For *Copecion* and *Ectocion* (which comprised three biozone groups), power was evaluated using Kruskal–Wallis tests, while for *Sifrhippus* (which comprised two biozone groups) power was evaluated using the Mann–Whitney *U*‐test. Simulations were performed using a priori (Fiel Peres 2026) moderate effect sizes (Ordinal eta‐squared = 0.06 for Kruskal–Wallis tests; Vargha–Delaney A = 0.64 for Mann–Whitney tests), and the minimum sample size required to achieve 80% statistical power at *α* = 0.05 was estimated.

Because high‐resolution stratigraphic data within the Cabin Forks locality of the Bighorn Basin for specimens of *S. sandrae* and *S. grangeri* were available (Secord et al. 2012), we were able to trace dental microwear across the PETM and the recovery interval for this lineage. Linear regressions were conducted that traced each DMTA variable across meter level within the Bighorn Basin, divided by the conclusion of the CIE at ~ meter level 46. This high‐resolution stratigraphy was not available for our sample of *Copecion* and *Ectocion*, precluding this type of analysis for these lineages (Gingerich 2003).

We employed quadratic discriminant analysis (QDA) to infer probable foraging guild membership for extinct ungulates based on DMTA variables, using modern African bovid foraging guilds as the training guide. Schwartz et al. (in press) developed a QDA model derived from the extant African bovid dataset presented in Scott et al. (2012) that predicts foraging guild from DMTA variables with 77.1% repeated 10‐fold cross‐validation accuracy. This established model was applied to the extinct taxa to generate specimen‐level posterior probabilities of guild membership.

To summarize foraging guild affinity, mean posterior probabilities were calculated for each Taxon—NALMA. Uncertainty in these mean estimates was quantified using nonparametric bootstrap resampling (5000 iterations) of specimen‐level posterior probabilities within each taxon, producing 95% confidence intervals (Table 3). This approach accommodates small sample sizes and the bounded, non‐normal distribution of posterior probabilities, providing a robust estimate of classification stability and foraging guild affinity. Principal component analysis (PCA) was then applied to the posterior probability matrix in order to visualize patterns of classification confidence and uncertainty. The model summarized posterior probability PCA space of extant taxon foraging guild using 95% confidence ellipses based on Mahalanobis distances (distance between a point and a probability distribution), while extinct taxa were projected onto the same space for comparative interpretation. Because the training data derive from extant ruminants rather than phylogenetically closer non‐ruminant herbivores, posterior probabilities should be interpreted as measures of similarity to modern guilds rather than direct dietary classifications. Consequently, the QDA is used here as a comparative framework for evaluating relative ecological affinities rather than assigning definitive diets. All analyzes were conducted using RStudio 2025.05.1 (RStudio Team 2025). The script used to conduct these analyzes and generate figures is available on Dryad (https://datadryad.org/share/LINK_NOT_FOR_PUBLICATION/JZpowabG_6UyXa_Ap0mCZR0‐am7EFXkN4IOltziYIa4).

## 3 Results

### 3.1 Dietary Foraging Guild

Dental microwear results for each taxon are presented in Table 1 and plotted alongside modern African bovids (Figure 3). Table 2 directly compares dental microwear of Paleocene–Eocene taxa with those of extant bovid foraging groups described in Scott et al. (2012). Overall, *Copecion*, *Ectocion*, and *Sifrhippus* consistently differ from obligate and variable grazers across *Asfc*, *epLsar*, *Smc*, and *Tfv*, while showing similarities with frugivores, browsers, and generalists.

**FIGURE 3:**
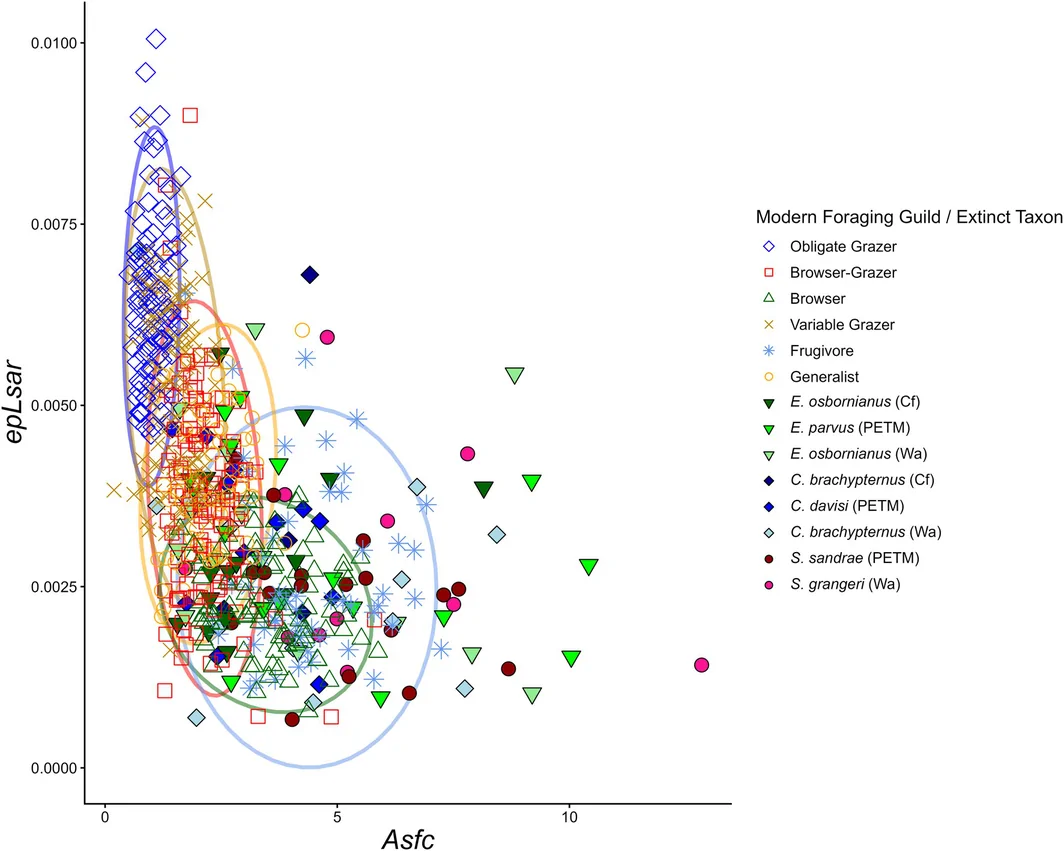
Bivariate scatterplot of dental microwear texture complexity (*Asfc*) and anisotropy (*epLsar*) with additional bovid data from Scott et al. (2012) and Schwartz et al. (in press). Data are plotted for individuals with foraging guild or Taxon—NALMA as indicated by the markers. Circles indicate 95% confidence intervals of foraging guilds from extant African bovids.

**TABLE 2:** Pairwise comparisons of taxa to modern bovid foraging guilds.

Taxon – NALMA	Variable	BROW	FRUG	GEN	B‐G	VG	OG
*C. brachypternus*_Cf_	*Asfc*	0.7805	0.7817	0.0529	**0.0456**	***p* < 0.001**	***p* < 0.001**
	*epLsar*	0.1991	0.2479	0.5863	0.7807	0.0661	**0.0031**
	*Smc*	***p* < 0.001**	0.5556	0.2882	0.0517	***p* < 0.001**	***p* < 0.001**
	*Tfv*	0.5937	0.2734	0.8373	0.1592	**0.0024**	***p* < 0.001**
	*HAsfc*_9×9_	0.2443	0.8964	0.8864	0.8811	0.3669	0.2197
*C. davisi*_PETM_	*Asfc*	0.4723	0.0729	0.0600	**0.0040**	***p* < 0.001**	***p* < 0.001**
	*epLsar*	0.0775	0.1410	0.1431	0.3362	***p* < 0.001**	***p* < 0.001**
	*Smc*	***p* < 0.001**	0.5836	0.1904	**0.0034**	***p* < 0.001**	***p* < 0.001**
	*Tfv*	0.0532	**0.0058**	0.5004	0.1487	***p* < 0.001**	***p* < 0.001**
	*HAsfc*_9×9_	***p* < 0.001**	0.2791	0.2436	**0.0456**	0.9287	0.5199
*C. brachypternus*_Wa_	*Asfc*	0.2658	**0.0330**	0.0953	0.0612	***p* < 0.001**	***p* < 0.001**
	*epLsar*	0.2364	0.3424	**0.0238**	0.0868	***p* < 0.001**	***p* < 0.001**
	*Smc*	***p* < 0.001**	0.5330	0.1904	**0.0015**	***p* < 0.001**	***p* < 0.001**
	*Tfv*	***p* < 0.001**	***p* < 0.001**	**0.0487**	0.8407	**0.0019**	***p* < 0.001**
	*HAsfc*_9×9_	***p* < 0.001**	0.0727	0.0522	**0.0036**	0.5480	0.9972
*E. osbornianus*_Cf_	*Asfc*	0.3832	**0.0447**	**0.0463**	**0.0255**	***p* < 0.001**	***p* < 0.001**
	*epLsar*	0.1061	0.1809	0.0513	0.1519	***p* < 0.001**	***p* < 0.001**
	*Smc*	***p* < 0.001**	0.6184	0.1783	**0.0017**	***p* < 0.001**	***p* < 0.001**
	*Tfv*	***p* < 0.001**	***p* < 0.001**	***p* < 0.001**	**0.0277**	0.3871	**0.0255**
	*HAsfc*_9×9_	**0.0050**	0.9059	0.8846	0.2632	0.2381	0.0795
*E. parvus* _PETM_	*Asfc*	0.7461	0.3682	***p* < 0.001**	***p* < 0.001**	***p* < 0.001**	***p* < 0.001**
	*epLsar*	0.0619	0.1350	**0.04521**	0.1503	***p* < 0.001**	***p* < 0.001**
	*Smc*	***p* < 0.001**	0.4025	0.0740	***p* < 0.001**	***p* < 0.001**	***p* < 0.001**
	*Tfv*	0.2201	**0.0254**	0.7515	**0.0045**	***p* < 0.001**	***p* < 0.001**
	*HAsfc*_9×9_	***p* < 0.001**	**0.0116**	**0.0060**	***p* < 0.001**	0.1927	0.5934
*E. osbornianus*_Wa_	*Asfc*	0.4356	0.0582	**0.0485**	**0.0281**	***p* < 0.001**	***p* < 0.001**
	*epLsar*	0.0602	0.1203	0.1145	0.2971	***p* < 0.001**	***p* < 0.001**
	*Smc*	***p* < 0.001**	0.3164	0.8361	0.1752	***p* < 0.001**	***p* < 0.001**
	*Tfv*	0.2031	**0.0288**	0.9200	**0.0211**	***p* < 0.001**	***p* < 0.001**
	*HAsfc*_9×9_	***p* < 0.001**	0.1311	0.1338	**0.0103**	0.8073	0.7818
*S. sandrae*_PETM_	*Asfc*	0.1400	0.9319	***p* < 0.001**	***p* < 0.001**	***p* < 0.001**	***p* < 0.001**
	*epLsar*	0.8136	0.9210	***p* < 0.001**	**0.0020**	***p* < 0.001**	***p* < 0.001**
	*Smc*	***p* < 0.001**	0.0654	**0.0039**	***p* < 0.001**	***p* < 0.001**	***p* < 0.001**
	*Tfv*	**0.0025**	***p* < 0.001**	0.1360	0.3330	***p* < 0.001**	***p* < 0.001**
	*HAsfc*_9×9_	0.7408	**0.0182**	**0.0168**	0.1195	***p* < 0.001**	***p* < 0.001**
*S. grangeri*_Wa_	*Asfc*	0.2649	0.9729	***p* < 0.001**	***p* < 0.001**	***p* < 0.001**	***p* < 0.001**
	*epLsar*	0.3410	0.4596	**0.0318**	0.0996	***p* < 0.001**	***p* < 0.001**
	*Smc*	***p* < 0.001**	0.1999	**0.0381**	***p* < 0.001**	***p* < 0.001**	***p* < 0.001**
	*Tfv*	***p* < 0.001**	***p* < 0.001**	**0.0143**	0.6031	**0.0351**	**0.0011**
	*HAsfc*_9×9_	**0.0038**	0.5911	0.5547	0.1803	0.5829	0.2521

*Note:* Benjamini–Hochberg Correction used. Bold indicates *p* < 0.05.

Abbreviations: B‐G, Browser‐Grazer Intermediate; BROW, Browser; FRUG, Frugivore; GEN, Generalist; OG, Obligate Grazer; VG, Variable Grazer.

*Copecion* exhibits the greatest similarity to browsers and frugivores, though differences from modern analogs are apparent across biozones. *C. brachypternus*_Cf_ differs primarily in *Asfc* and *Smc*, whereas *C. davisi*_PETM_ differs in *Asfc* and *Smc*, as well as *Tfv* and *HAsfc*_9×9_. *C. brachypternus*_Wa_ exhibits differences in all DMTA variables, with each foraging guild differing in at least one variable. It is important to note that the small sample size (*n* = 4) of *C. brachypternus*_Cf_ instills little confidence in assignment of foraging guild affinity.

*Ectocion* similarly shows the greatest overlap with browsing, frugivorous, and generalist foraging guilds, although *Tfv* represents a notable source of differentiation among species. *E. osbornianus*_Cf_ differs from several guilds in *Asfc*, *Smc*, and *HAsfc*_9×9_, with *Tfv* differing from nearly all guilds except variable grazers (Table 2). *E. parvus* _PETM_ and *E. osbornianus*_Wa_ share similar patterns, characterized by elevated *Asfc*, reduced *Smc* relative to browsers and browser‐grazers, and lower *HAsfc*_9×9_ compared with non‐grazing guilds.

Both species of *Sifrhippus* show similar patterns of overlap with browsers and frugivores while maintaining distinction from grazers. Relative to modern foraging guilds, *S. sandrae*_PETM_ and *S. grangeri*_Wa_ are primarily separated by differences in *Asfc*, *epLsar*, *Smc*, and *Tfv*. *HAsfc*_9×9_ further differentiates *S. sandrae*_PETM_ from most modern guilds, whereas *S. grangeri*_Wa_ only differs significantly from browsers for this variable.

Our QDA model recovered varying levels of support for the inferred foraging affinities of the extinct ungulates (Table 3; Figure 4). Mean posterior probabilities and their associated bootstrap 95% confidence intervals indicate that *S. grangeri*_Wa_ (70.3%), *S. sandrae*_PETM_ (62.4%), *C. brachypternus*_Wa_ (57.0%), and *E. parvus* _PETM_ (51.1%) show the strongest support for affinity with the modern frugivore guild. Both *C. davisi*_PETM_ and *E. osbornianus*_Wa_ also exhibited their highest posterior probabilities for frugivores, although with greater overlap among alternative guilds as reflected by their broader bootstrap confidence intervals. The remaining taxa showed diffuse posterior probability distributions spanning multiple guilds, indicating weaker support for any single modern ecological analog.

**TABLE 3:** Results of quadratic discriminant analysis showing the two highest mean posterior probabilities for foraging guild affinity for each taxon, arranged by North American Land Mammal Age (NALMA).

Taxon – NALMA	*n*	Greatest affinity	$\bar{P}$_post_(%)	95% CI
*C. brachypternus*_Cf_	4	Generalist	35.9	0.4–71.35
		Browser	35.3	3.09–74.04
*C. davisi*_PETM_	12	Frugivore	49.0	25.71–71.84
		Generalist	24.5	9.78–40.33
*C. brachypternus*_Wa_	14	**Frugivore**	57.0	35.23–77.86
		Variable Grazer	22.3	6.02–42.23
*E. osbornianus*_Cf_	15	Browser‐Grazer	39.2	25.39–54.22
		Frugivore	25.9	11.08–44.04
*E. parvus* _PETM_	18	**Frugivore**	51.1	31.32–71.36
		Generalist	29.1	11.78–47.96
*E. osbornianus*_Wa_	14	Frugivore	29.9	8.59–51.99
		Generalist	25.4	6.78–45.12
*S. sandrae*_PETM_	18	**Frugivore**	62.4	43.63–80.19
		Browser‐Grazer	18.1	5.18–34.02
*S. grangeri*_Wa_	11	**Frugivore**	70.3	46.77–90.56
		Generalist	11.0	0–28.2

*Note:* Mean posterior probabilities greater than 50% indicated in bold. 95% confidence interval (95% CI) determined using nonparametric bootstrap resampling (5000 iterations).

Abbreviations: B‐G, Browser‐Grazer Intermediate; BROW, Browser; FRUG, Frugivore; GEN, Generalist; OG, Obligate Grazer; VG, Variable Grazer.

**FIGURE 4:**
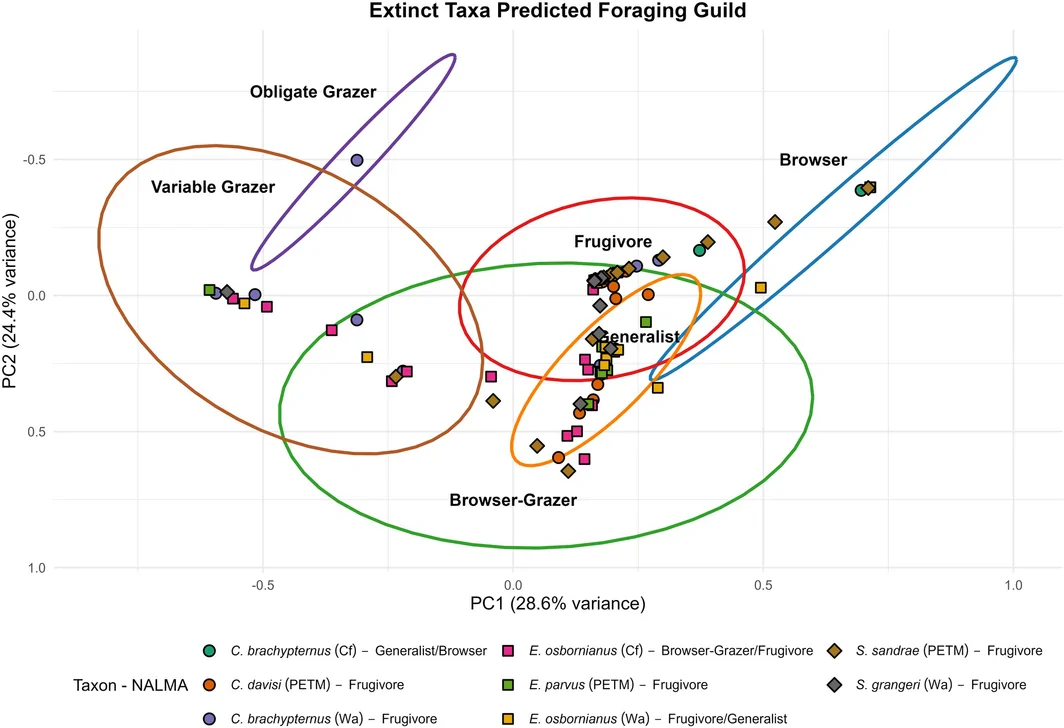
Plot of quadratic discriminant analysis on principal components with 95% probability ellipses using Mahalanobis distances based on model in Schwartz et al. (in press). Foraging guilds with the highest mean posterior probability were labeled next to each taxon. Taxa lacking clear separation between the first‐ and second‐ranked guilds were interpreted as having ambiguous affinities.

### 3.2 Differences Between Contemporaneous Taxa

In the Clarkforkian NALMA, only *E. osbornianus*_Cf_ and *C. brachypternus*_Cf_ were contemporaneous in the Bighorn Basin. Interestingly, only textural fill volume differed significantly, being higher (*p* = 0.0278) in *C. brachypternus*_Cf_ compared to *E. osbornianus*_Cf_ (Table 4; Figure 5). No other dental microwear variables showed significant differences.

**TABLE 4:** North American Land Mammal Age (NALMA) species pairwise comparisons.

Taxon – NALMA		*Asfc*	*epLsar*	*Smc*	*Tfv*	*HAsfc*_9×9_
*C. brachypternus*_Cf_	*E. osbornianus*_Cf_	0.2301	0.7642	0.5766	**0.0278**	1.00
*C. davisi*_PETM_	*E. parvus* _PETM_	0.2020	0.6586	0.9677	0.8394	0.2894
*C. davisi*_PETM_	*S. sandrae*_PETM_	**0.0252**	0.2084	**0.0035**	0.8107	**0.0326**
*E. parvus* _PETM_	*S. sandrae*_PETM_	0.2027	0.1869	**0.0023**	0.5204	***p* < 0.001**
*C. brachypternus*_Wa_	*E. osbornianus*_Wa_	0.9603	1.0000	**0.0499**	0.2239	0.8423
*C. brachypternus*_Wa_	*S. grangeri*_Wa_	0.2258	0.9876	0.1341	0.4011	0.6454
*E. osbornianus*_Wa_	*S. grangeri*_Wa_	0.4133	0.8010	**0.0014**	0.0850	0.9882

*Note:* Benjamini–Hochberg Correction, comparison between *C. brachypternus*_Cf_ and *E. osbornianus*_Cf_ data derived from Mann–Whitney *U*‐test. Bold indicates *p* < 0.05.

**FIGURE 5:**
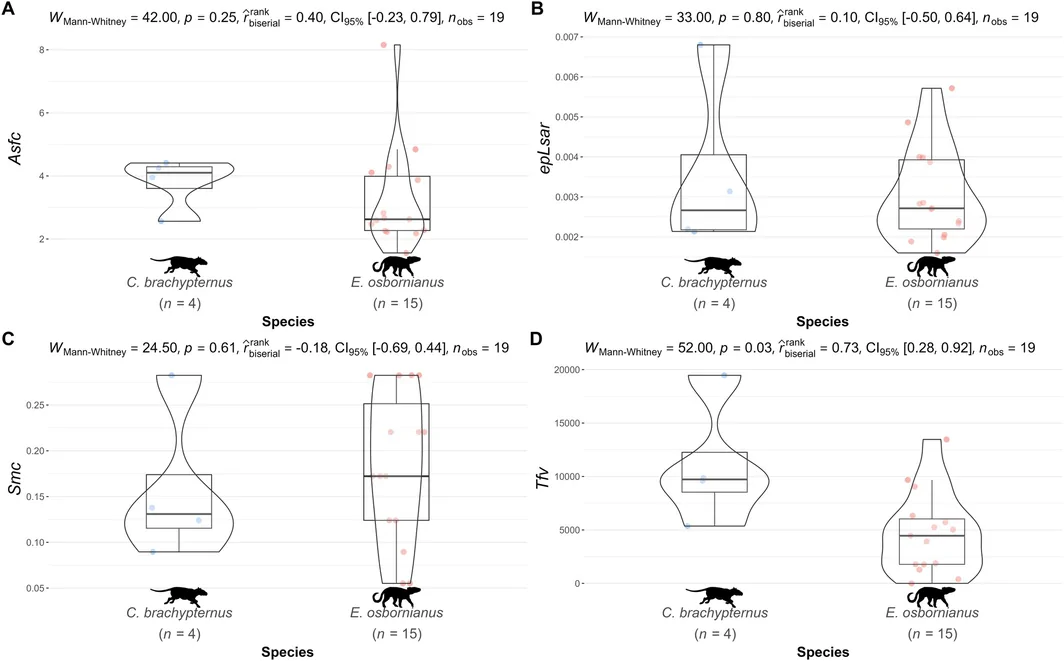
Results of Mann–Whitney *U*‐test for dental microwear texture analysis of *Copecion brachypternus* and *Ectocion osbornianus* from the Clarkforkian North American Land Mammal age (NALMA). (A) complexity (*Asfc*), (B) anisotropy (*epLsar*), (C) scale of maximum complexity (*Smc*), and (D) textural fill volume (*Tfv*). Boxes represent the 25th–75th interquartile range. Whiskers extend to 1.5 times the interquartile range. Violin outlines represent smoothed kernel density estimates of the distribution. Silhouette of *Ectocion* provided by Colin McElroy (vectorized by Andrew Schwartz) under CC BY 4.0.

While *C. davisi*_PETM_ and *E. parvus* _PETM_ did not differ significantly in dental microwear, both showed significant differences with *S. sandrae*_PETM_ (Table 4; Figure 6). *S. sandrae*_PETM_ exhibited significantly higher complexity (*p* = 0.0252) and heterogeneity of complexity (*p* = 0.0326), as well as lower scale of maximum complexity (*p* = 0.0035) than *C. davisi*_PETM_ (Figure 6). In comparison to *E. parvus* _PETM_, *S. sandrae*_PETM_ exhibited significantly higher heterogeneity of complexity at (*p* < 0.001) and lower scale of maximum complexity (*p* = 0.0023).

**FIGURE 6:**
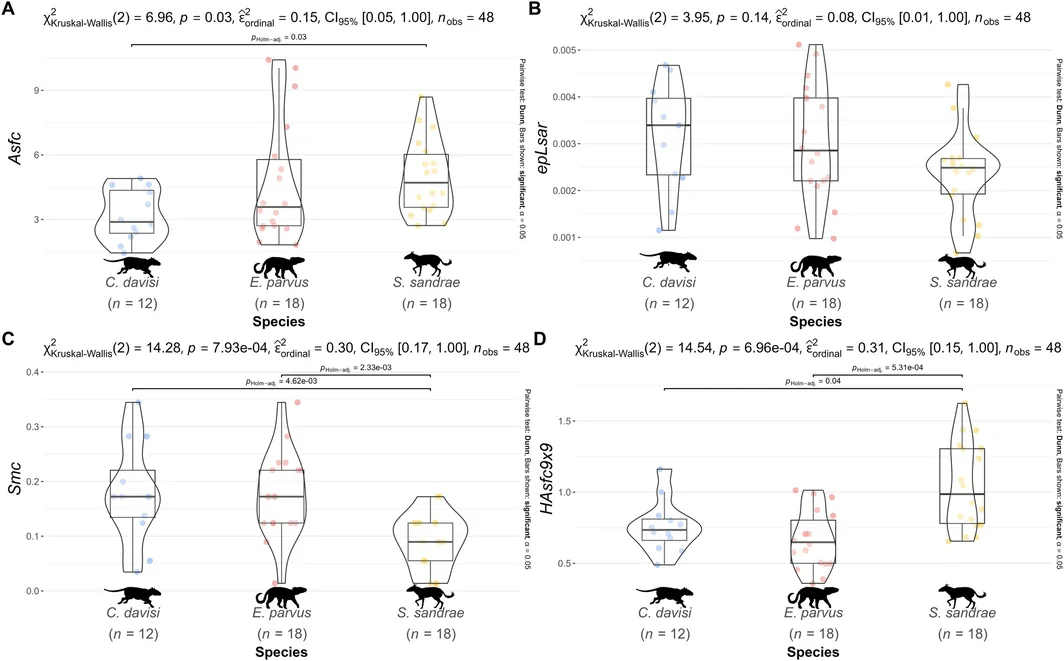
Results of Kruskal–Wallis test for dental microwear texture analysis of *Copecion davisi*, *Ectocion parvus*, and *Sifrhippus sandrae* from the Paleocene—Eocene Thermal Maximum (PETM). Significant differences between (A) complexity (*Asfc*), (B) anisotropy (*epLsar*), (C) scale of maximum complexity (*Smc*), and (D) heterogeneity of complexity at 9 × 9 scale (*HAsfc*_9×9_) adjusted using Benjamini Hochberg procedure. Boxes represent 25th–75th interquartile range. Whiskers extend to 1.5 times the interquartile range. Violin outlines represent smoothed kernel density estimates of the distribution. Silhouette of *Ectocion* provided by Colin McElroy (vectorized by Andrew Schwartz) under CC BY 4.0.

Following the PETM, the early Wasatchian marks the return of *C. brachypternus*_Wa_ and *E. osbornianus*_Wa_, as well as the first appearance of *S. grangeri*_Wa_. Interestingly, few significant differences in dental microwear were recovered between these three contemporaneous taxa (Table 4; Figure 7). These differences are limited to scale of maximum complexity, which is higher in *E. osbornianus*_Wa_ compared to both *C. brachypternus*_Wa_ (*p* = 0.0499) and *S. grangeri*_Wa_ (*p* = 0.0014).

**FIGURE 7:**
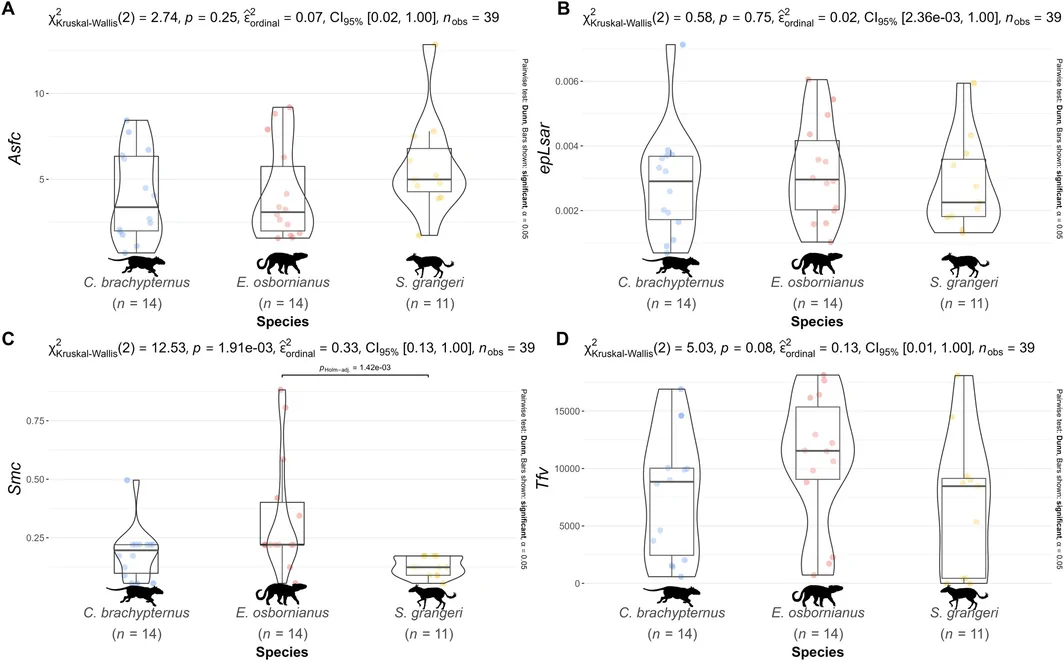
Results of Kruskal–Wallis test for dental microwear texture analysis of *Copecion brachypternus*, *Ectocion osbornianus*, and *Sifrhippus grangeri* following the Paleocene–Eocene Thermal Maximum (PETM) in the early Eocene. Significant differences between (A) complexity (*Asfc*), (B) anisotropy (*epLsar*), (C) scale of maximum complexity (*Smc*), and (D) textural fill volume (*Tfv*) adjusted using Benjamini Hochberg procedure. Boxes represent 25th–75th interquartile range. Whiskers extend to 1.5 times the interquartile range. Violin outlines represent smoothed kernel density estimates of the distribution. Silhouette of *Ectocion* provided by Colin McElroy (vectorized by Andrew Schwartz) under CC BY 4.0.

### 3.3 Differences Across the PETM

Differences in dental microwear were traced within lineages over time from the Clarkforkian NALMA through the PETM into the Wasatchian NALMA (Table 5). For *Copecion*, no significant differences were recovered in the studied time interval, with each variable having negligible effect sizes, approximately zero effect size of all variables. Power analysis estimated that approximately 240 total specimens distributed proportionally among groups (Cf = 32, PETM = 112, Wa = 96) would be required to detect a moderate temporal dietary shift (*ε* ^2^ = 0.06) with ≥ 80% power using a Kruskal–Wallis test.

**TABLE 5:** Lineage‐specific pairwise comparisons over time; Benjamini–Hochberg correction, *Sifrhippus* comparison derived from Mann–Whitney *U*‐test.

Variable	Taxon – NALMA	*C. davisi*_PETM_	*C. brachypternus*_Wa_
*Asfc*	*C. brachypternus*_Cf_	1.0000	0.8412
	*C. davisi*_PETM_		0.9713
*epLsar*	*C. brachypternus*_Cf_	0.7430	1.0000
	*C. davisi*_PETM_		0.8833
*Tfv*	*C. brachypternus*_Cf_	0.7679	1.0000
	*C. davisi*_PETM_		0.7053
*Smc*	*C. brachypternus*_Cf_	0.9821	1.0000
	*C. davisi*_PETM_		0.9751
*HAsfc*_9×9_	*C. brachypternus*_Cf_	0.7183	0.9914
	*C. davisi*_PETM_		0.5739

		*E. parvus*_PETM_	*E. osbornianus*_Wa_
*Asfc*	*E. osbornianus*_Cf_	0.2719	0.5560
	*E. parvus* _PETM_		0.4433
*epLsar*	*E. osbornianus*_Cf_	0.9510	1.0000
	*E. parvus* _PETM_		1.0000
*Smc*	*E. osbornianus*_Cf_	0.7748	0.0985
	*E. parvus* _PETM_		0.0834
*Tfv*	*E. osbornianus*_Cf_	**0.0046**	**0.0051**
	*E. parvus* _PETM_		0.7148
*HAsfc*_9×9_	*E. osbornianus*_Cf_	0.2140	0.2146
	*E. parvus* _PETM_		0.8088

		*S. grangeri*_Wa_
*Asfc*	*S. sandrae*_PETM_	0.5292
*epLsar*	*S. sandrae*_PETM_	0.5896
*Smc*	*S. sandrae*_PETM_	0.0914
*Tfv*	*S. sandrae*_PETM_	0.3685
*HAsfc*_9×9_	*S. sandrae*_PETM_	0.0533

*Note:* Bold indicates *p* < 0.05.

*E. osbornianus*_Cf_ showed significantly lower textural fill volume when compared to both *E. parvus* _PETM_ (*p* = 0.005) and *E. osbornianus*_Wa_ (*p* = 0.005) (Figure 8). The effect size for textural fill volume was over three times larger (0.382) than the next highest variable effect size (Smc = 0.128). To detect a moderate effect size for *Asfc*, *epLsar*, *Smc*, and *HAsfc*_9×9_, power analysis estimated that approximately 106 total specimens distributed proportionally among groups (Cf = 34, PETM = 32, Wa = 40) to achieve ≥ 80% power using a Kruskal–Wallis test.

**FIGURE 8:**
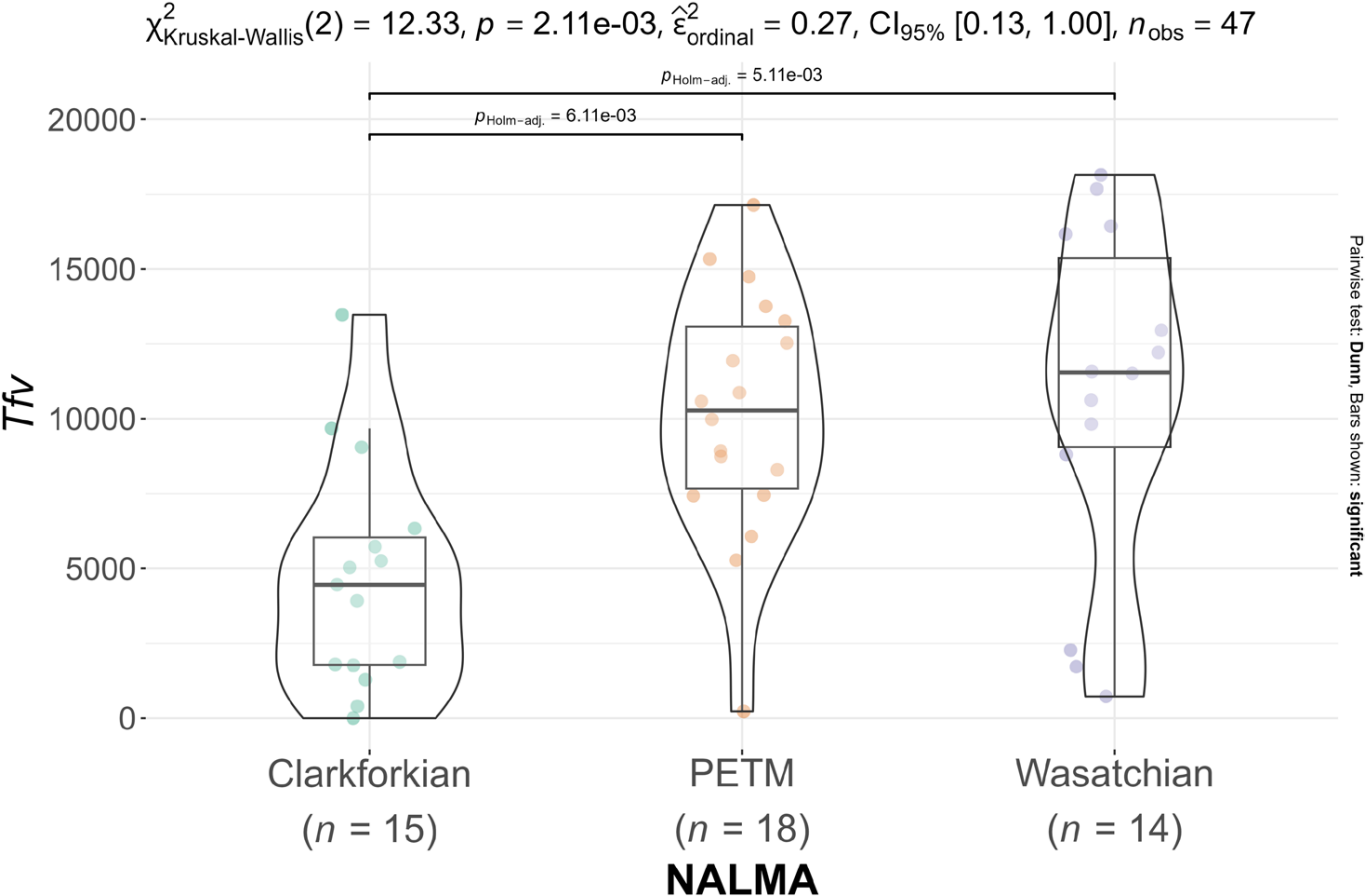
Results of Kruskal–Wallis test for textural fill volume (*Tfv*) change through the Paleocene–Eocene Thermal Maximum (PETM) in *Ectocion*. Significant differences adjusted using Benjamini Hochberg procedure. Boxes represent 25th–75th interquartile range. Whiskers extend to 1.5 times the interquartile range. Violins outlines represent smoothed kernel density estimates of the distribution.

No significant differences in dental microwear were recovered between *S. sandrae*_PETM_ and *S. grangeri*_Wa_, though power analysis estimated that approximately 145 total specimens distributed proportionally among groups (PETM = 55, Wa = 90) would be required to detect a moderate effect (VDA ≥ 0.64) with ≥ 80% power using a Mann–Whitney *U*‐test.

Tracing dental microwear specifically across the PETM body and recovery in the Cabin Forks locality of the Bighorn Basin also revealed no significant DMTA trends in the *Sifrhippus* lineage as the hyperthermal progressed and recovered (Table 6; Figure 9). Only *Asfc* approaches significance (*R* ^2^ = 0.16; *p* = 0.10) in *S. sandrae*_PETM_. Three outlying data points for *Asfc* (two prior to and one following the PETM) and two for *epLsar* (both following the PETM) were evaluated using Cook's distance to assess their influence on the fitted regression models. Sensitivity analyzes indicated that excluding the flagged *Asfc* observations modestly altered the estimated slopes but did not change the overall statistical conclusions; the relationships remained nonsignificant both prior to (*p* = 0.093) and following the PETM (*p* = 0.376). For *epLsar*, excluding the two flagged observations reduced the estimated slope and explained variance, but the relationship also remained statistically nonsignificant (*p* = 0.463, compared with *p* = 0.133 in the original model). Thus, although the flagged observations influenced the estimated slopes and explanatory power of the regression models, their exclusion did not alter the overall conclusion that none of the relationships was statistically significant.

**TABLE 6:** Results of linear regressions for *S. sandrae* during the Body of the PETM and *S. grangeri* during the Recovery interval (early Wasatchian).

Taxon	PETM	Variable	Linear regression (*Y* =)	*R* ^2^	*p*
*S. sandrae*	Body	*Asfc*	−0.073*x* + 7.24	0.16	0.0997
		*epLsar*	1.92 × 10^−5^ *x* + 0.002	0.042	0.415
		*Smc*	0.001*x* + 0.0574	0.0471	0.387
		*Tfv*	114*x* + 4950	0.0368	0.446
		*HAsfc*_9×9_	8.27 × 10^−4^ *x* + 1.02	0.00067	0.919
*S. grangeri*	Recovery	*Asfc*	−0.032*x* + 8.02	0.00867	0.785
		*epLsar*	8.28 × 10^−5^ *x* + 0.003	0.232	0.133
		*Smc*	−0.001*x* + 0.183	0.0265	0.632
		*Tfv*	51.6*x* + 3160	0.00486	0.839
		*HAsfc*_9×9_	0.004*x* + 0.547	0.00976	0.773

**FIGURE 9:**
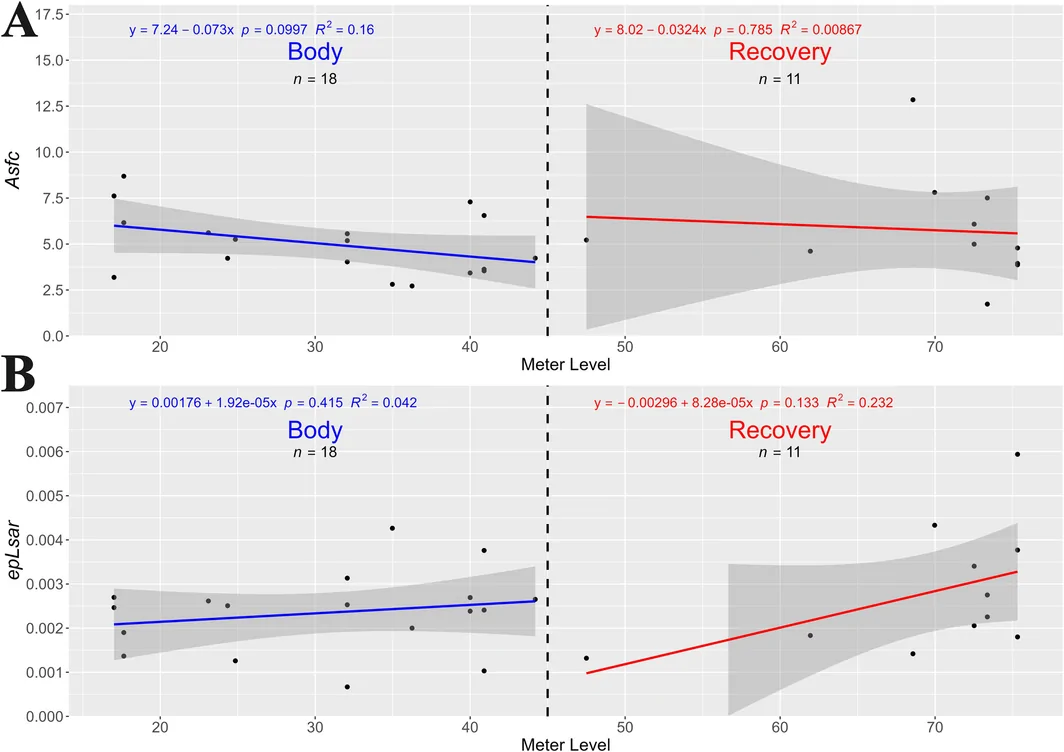
Linear regressions of dental microwear change over the Paleocene—Eocene Thermal Maximum (PETM) in *Sifrhippus sandrae* (left) and *Sifrhippus grangeri* (right); (A) complexity (*Asfc*), (B) anisotropy (*epLsar*). Separation between “Body” and “Recovery” intervals of the PETM demarcated by dotted vertical line at meter level ~46.

## 4 Discussion

Archaic ungulates from the Paleocene–early Eocene of the Bighorn Basin show generally similar dental microwear across taxa that are broadly consistent with frugivory. Dental microwear from these ancient ungulates reflects considerable niche overlap combined with minimal dietary ecological change over time across the PETM (Table 5). Similarities to modern browsing, frugivorous, and generalist foragers were not unexpected outcomes based on tooth morphology and the floral communities of North America during this time (Figure 3). From the overall similar dental morphology of these taxa, crushing and pulping of relatively hard objects (particularly fruit and seeds) with occasional shearing of tough foliage is inferred, congruent with the reconstructed floral ecosystems for North America at the Paleocene–Eocene boundary (Korasidis and Wing 2023). In the Bighorn Basin, early Cenozoic forests were generally temperate and subtropical, with conifers, ferns, and eudicots (particularly abundant with species of Fabaceae). Although grasses were present in these environments (having evolved by the Lower Cretaceous), vast grasslands would not become ubiquitous in North America until the following Oligocene and Miocene epochs (Strömberg 2011; Wu et al. 2018). It is therefore unsurprising that all taxa exhibit significant differences in most dental microwear variables compared to variable grazers, obligate grazers, and browser‐grazer intermediates (Table 2).

Comparison of our data to that of the extant African bovid dataset allows some confidence toward reconstruction of foraging behavior for each extinct taxon. Using Kruskal–Wallis tests and our QDA model, we were able to identify foraging guild affinity through direct comparison and mean posterior probabilities for each taxon based on dental microwear (Tables 2, 3). *C. brachypternus*_Cf_ closely approximates frugivores and generalists based on DMTA variables, while mean posterior probabilities are roughly equal between generalist (35.9%) and browser (35.3%), though our diminutive sample size (*n* = 4) instills little confidence. *C. davisi*_PETM_ is most similar to browsers, frugivores, and generalists, while mean posterior probabilities show greatest affinities to frugivores (49.0%) and generalists (24.5%). *C. brachypternus*_Wa_ exhibits numerous pairwise differences with all guilds but mean posterior probabilities show greatest affinity to frugivores (57.0%). These comparisons suggest that species within this lineage exhibited the greatest similarity to modern mixed feeders with relatively high fruit consumption, although these ecological affinities should not be interpreted as direct evidence that identical diets were consumed.

*E. osbornianus*_Cf_ most closely approximates browsers based on complexity and anisotropy, while also showing similarity to frugivores and browser‐grazers based on anisotropy (Table 2). Notably, *E. osbornianus*_Cf_ only shows similar textural fill volume to variable grazers. *E. osbornianus*_Cf_ exhibits mixed affinities through low mean posterior probabilities to browser‐grazers (39.2%) and frugivores (25.9%). *E. parvus* _PETM_ also showed pairwise comparison similarities to browsers based on anisotropy, complexity and textural fill volume, as well as similarities to frugivores based on anisotropy, complexity, and scale of maximum complexity (Table 2). Unlike *E. osbornianus*_Cf_, mean posterior probabilities show greatest affinity to frugivores (51.1%) in *E. parvus* _PETM_. Like *E. osbornianus*_Cf_ and *E. parvus* _PETM_, *E. osbornianus*_Wa_ too shows pairwise similarities with browsers and frugivores. However, mean posterior probabilities are low in this taxon, with greatest affinities to frugivores (29.9%) and generalists (25.4%).

Both species of *Sifrhippus* exhibit considerable pairwise similarities to browsers and frugivores (Table 2). *S. sandrae*_PETM_ shows greatest similarity to browsers and frugivores based on complexity and anisotropy while mean posterior probabilities show greatest affinity to frugivores (62.4%; Table 3). In a similar vein, *S. grangeri*_Wa_ most closely resembles browsers and frugivores based on pairwise comparisons, while mean posterior probabilities similarly show greatest affinity to frugivores (70.3%).

Heterogeneity of complexity was often incongruent with foraging guild suggested by pairwise comparisons of complexity, anisotropy, scale of maximum complexity, and textural fill volume. This likely drove much of the ambiguity of our QDA toward assigning foraging guild affinities based on mean posterior probabilities. With the exception of *S. sandrae*_PETM_, all taxa did not show significant differences compared to variable and obligate grazers in terms of heterogeneity of complexity (Table 2), likely indicating similar patterns of wear features across adjoining surfaces resulting from diversity of wear‐causing particles (Krueger et al. 2008). Some studies hypothesize that food material diversity is positively correlated with heterogeneity of complexity, suggesting that more opportunistic and dietarily broad species are expected to have greater heterogeneities of complexity than species with more specialized diets (Scott et al. 2012; Souron et al. 2015). Among African bovids, Scott et al. (2012) found that both variable and obligate grazers exhibit the lowest heterogeneity of complexity while browsers, frugivores, and generalists tend to have higher values. One possible interpretation is that while *Copecion*, *Ectocion*, and *S. grangeri*_Wa_ habitually consumed herbaceous vegetation and fruit, the diversity of material properties in these foods was relatively homogenous, like that of modern grasses.

That only *S. sandrae*_PETM_ exhibits higher heterogeneity of complexity than both predominant grazing guilds implies that this taxon consumed a greater variety of wear‐causing particles. It is worth noting that *S. sandrae*_PETM_ is the only PETM immigrant taxon into the Bighorn Basin within our dataset. More opportunistic foraging behavior in response to this new environment is one possibility as to why heterogeneity of complexity is so high in this taxon. However, the relationship between heterogeneity of complexity and diet needs to be further clarified to better understand how this precisely relates to foraging ecology.

A notable peculiarity within our results is the large number of early ungulate specimens that exhibit considerably higher complexity values than those represented by extant African bovids (Figure 3). If interpreted as a genuine dietary signal, these values suggest that many of these individuals consumed harder foods than those typically consumed by bovids, including frugivorous taxa (the guild exhibiting the highest *Asfc* values). In fact, these data compare favorably with those reported for the hard‐object feeding tufted capuchin ( *Sapajus apella* ) by Scott et al. (2005). This primate is known to consume hard, brittle foods such as palm nuts (Janson and Boinski 1992; Terborgh 1983). While we refrain from interpreting these similarities as strong evidence for hard nut or seed consumption by early ungulates, the absence of hard‐object‐specialized bovids in our comparative dataset may contribute to the “outlying” complexity values observed in these specimens. Future work should investigate differences in dental microwear between fruit and hard nut or seed consumption in ungulates to better contextualize the potential for hard nut or seed feeding among early ungulates.

Further driving ambiguity in reconstructing foraging guilds are the considerable floral differences between early Cenozoic North America and modern ecosystems. As noted previously, grasses were either extremely rare or absent during this time (Crepet and Feldman 1991). Therefore, dental microwear similarities to modern grazers may reflect consumption of plants with similar material properties to grass. This possibility is particularly relevant for ferns and horsetails, which were prominent components of Bighorn Basin floras (Korasidis and Wing 2023; Wing and Currano 2013) but are poorly represented in studies of mammalian dietary ecology. Given the relatively high rigidity of fern leaves and exceptional stiffness of horsetail stems, these plants could plausibly produce microwear textures that overlap with those of mechanically resistant vegetation represented in modern DMTA datasets. In particular, the high bending elastic modulus of horsetail stems raises the possibility that similarities to modern grazers in some early ungulates could reflect habitual consumption of horsetail stems rather than grasses. However, without experimental data linking fern and horsetail mastication to specific dental microwear textures, this possibility cannot currently be distinguished from other dietary explanations. Experimental studies examining the effects of fern and horsetail consumption on dental microwear are therefore needed to improve dietary reconstruction in ancient ecosystems lacking close modern floral analogs.

An additional source of uncertainty arises from the predictive performance of the QDA model itself. Although the classifier achieved an overall repeated 10‐fold cross‐validation accuracy of 77.1% (Schwartz et al. in press), approximately one‐quarter of extant specimens were assigned to an incorrect foraging guild, reflecting overlap in DMTA textures among modern foraging guild categories. Consequently, confidence in reconstructed ecological affinities should be interpreted alongside the posterior probability distributions and their bootstrap confidence intervals. While some taxa exhibit high posterior probability distributions and relatively narrow bootstrap confidence intervals (*S. grangeri*_Wa_ posterior probability = 70.3%; CI: 46.77%–90.56%), others express low posterior probabilities and wide bootstrap confidence intervals (Table 3), highlighting ambiguities in guild affinity partly due to small sample sizes. Taxa exhibiting diffuse posterior probabilities or substantial overlap among guilds warrant more cautious interpretation than those consistently assigned high posterior support for a single guild.

A second limitation of this study is that the comparative framework relies on extant African bovids rather than phylogenetically closer non‐ruminant ungulates. Although bovids and equids share a predominantly transverse chewing stroke and similar occlusal geometry (Kelly et al. 2021; Mihlbachler et al. 2016), differences in masticatory mechanics and digestive physiology likely influence how dental microwear records diet. As argued by Mihlbachler et al. (2016), phylogenetic history and chewing behavior can contribute to microwear variation independently of diet. Consequently, the posterior probabilities generated by our QDA should be interpreted as probabilistic ecological affinities rather than definitive dietary assignments. They identify the modern foraging guilds whose microwear textures most closely resemble those of the extinct taxa but cannot establish that identical foods or feeding strategies produced those textures.

Lastly, DMTA is unfit to evaluate hypotheses related to omnivory within these taxa. While omnivory is plausible for many groups of basal mammals including archaic ungulates (Grossnickle and Newham 2016), incipient lophodonty combined with the sheer abundance of individuals of these taxa during the Paleocene–early Eocene within the Bighorn Basin suggests herbivory to be more likely (Collinson and Hooker 1991; Thewissen 1990). Nevertheless, omnivory should be considered when attempting to reconstruct the paleoecology of early ungulates. Although DMTA can distinguish diet based on material properties, it is unable to differentiate between plant and animal tissue consumption. Instead, future studies should pair these DMTA data with stable nitrogen and/or calcium isotope analysis of fossil ungulate teeth to infer the degree to which these taxa were omnivorous (Clementz et al. 2003; Leichliter et al. 2023; Melin et al. 2014).

Taken together, these sources of uncertainty indicate that our conclusions are strongest at the level of broad ecological affinity rather than precise reconstruction of dietary composition. Despite these limitations, the combination of pairwise DMTA comparisons, posterior probability distributions, and bootstrap confidence intervals provides a robust framework for evaluating relative dietary similarity among extinct taxa while explicitly acknowledging uncertainty.

Future work should focus on developing a comprehensive dental microwear comparative dataset for extant perissodactyls (Equidae, Rhinocerotidae, and Tapiridae) spanning a broad range of foraging ecologies to complement the African bovid dataset of Scott et al. (2012). Although such a dataset will be challenging to assemble because of the limited diversity and ecological breadth of living perissodactyls compared to bovids (Cifelli 1981), it would substantially improve the phylogenetic appropriateness of comparative analyzes and allow the ecological hypotheses presented here to be tested more rigorously.

There are notable similarities in dental microwear between sympatric taxa. During the Clarkforkian NALMA, *E. osbornianus*_Cf_ and *C. brachypternus*_Cf_ exhibited considerably similar dental microwear. These two species of phenacodontid were relatively similar in size and morphology, hinting at direct competition for niche space. Differences in dental morphology show incipient lophodonty in *Ectocion*, suggesting superior shearing capacity in comparison to *Copecion*, possibly resulting in a competitive advantage in the browser niche for the former. Notably, *E. osbornianus*_Cf_ is far more common in the Bighorn Basin than *C. brachypternus*_Cf_ (Gingerich 2003). Both lineages are similarly abundant during the PETM and early Wasatchian, despite the immigration of new herbivores into the Bighorn Basin including *Sifrhippus* and the early artiodactyl *Diacodexis* (Gingerich 1989; Rose 1981).

Higher textural fill volume in *C. brachypternus*_Cf_ than in *E. osbornianus*_Cf_ is notable as the only DMTA variable in which these taxa differ significantly (Table 4, Figure 5). Typically, textural fill volume is correlated with complexity and is indicative of hard, brittle object consumption (Desantis et al. 2017). Exhibiting higher textural fill volume without the corresponding higher complexity is therefore perplexing. Textural fill volume recovered for *C. brachypternus*_Cf_ is consistent with browsing and frugivory, while *E. osbornianus*_Cf_ has values that most closely approximate variable grazing (exhibiting fewer deep pits than expected). Scott et al. (2012) postulated that greater textural fill volume is indicative of larger amounts of dental microwear, particularly in response to mastication of higher quantities of large abrasives. Desantis and Haupt (2014) suggest that differences in textural fill volume may actually be related to the coarseness of objects consumed. It is therefore plausible that *E. osbornianus*_Cf_ incorporated smaller quantities of abrasives or less‐coarse foods than *C. brachypternus*_Cf_, though it is unclear at this time.

During the PETM, *S. sandrae*_PETM_ immigrated into the Bighorn Basin, joining the phylogenetically dwarf *C. davisi*_PETM_ and *E. parvus* _PETM_. Sympatric taxa during the PETM show significantly different dental microwear, with *S. sandrae*_PETM_ having lower scale of maximum complexity and higher heterogeneity of complexity than both *C. davisi*_PETM_ and *E. parvus* _PETM_, as well as higher complexity compared to *C. davisi*_PETM_ (Table 4, Figure 6). Higher complexity is generally indicative of hard‐object consumption, typically in the form of fruits, seeds, and nuts (Scott 2012; Scott et al. 2005), while greater scale of maximum complexity suggests less wear at very fine scales and/or more wear at coarser scales, usually being lowest in frugivores and generalists (Scott 2012). Based on dental morphology alone, these results are unexpected. While the “bunolophodont” dentition of early equids like *Sifrhippus* suggests considerable frugivory, incipient crest formation between cusps seems to be adaptations for shearing tough, crack‐resistant leaves. No significant differences in anisotropy between these taxa are therefore perplexing.

That differences in dental microwear between *E. parvus* _PETM_ and *S. sandrae*_PETM_ are limited to scale of maximum complexity and heterogeneity of complexity is also intriguing. Unlike *Copecion*, both genera possessed adaptations for vertical shearing, with *Sifrhippus* also emphasizing transverse movement (Radinsky 1966; Rensberger 1986). Based on this morphology, it may be inferred that *Sifrhippus* was adapted for greater processing of food during mastication (Hiiemae and Crompton 1985). While lower scale of maximum complexity values in *S. sandrae*_PETM_ may be suggestive of greater frugivory, greater heterogeneity of complexity may reflect a larger diversity of foods. Notably, *E. parvus* _PETM_ was of similar size to *C. davisi*_PETM_ and would likely have had similar gastrointestinal complexity (Gingerich 2003). Therefore, similar explanations for differences between *S. sandrae*_PETM_ and *C. davisi*_PETM_ may also apply to *E. parvus* _PETM_.

Following the PETM, all three lineages rapidly increased in body mass to pre‐PETM values or larger as in the case of *S*. *grangeri*_Wa_ (Gingerich 2006; Secord et al. 2012). Notably, only scale of maximum complexity differs significantly across taxa, being highest in *E*. *osbornianus*_Wa_ (Table 4, Figure 7). Because this is indicative of lesser amounts of “fine‐scale” wear, it is possible that this represents consumption of foods possessing fewer fine‐scale particles or perhaps lesser contact with exogenous grit particles.

Overall, these results indicate substantial dietary overlap among sympatric taxa, with relatively few significant differences in dental microwear despite differences in body size and dental morphology. Because DMTA primarily records the mechanical properties of foods consumed over the last days to weeks of life (Winkler, Schulz‐Kornas, et al. 2020), it has limitations regarding differentiating microhabitats or community assembly processes. Consequently, our results demonstrate broad similarity in dietary mechanics but cannot, by themselves, identify the ecological processes that permitted coexistence.

The extensive dietary overlap reconstructed here among closely related taxa of comparable size and morphology is nevertheless intriguing, as phylogenetic niche conservatism predicts intensive competition for resources among closely‐related species (Cavender‐Bares et al. 2009; Mayfield and Levine 2010; Ricklefs 2010). One ecological explanation is niche unsaturation within the Bighorn Basin during the PETM and early Eocene. Unsaturated communities may arise following abiotic perturbations that reduce population densities or contract niche space through body size reduction, both of which characterized mammalian communities during the PETM (Gingerich 2006; Mcinerney and Wing 2011; Secord et al. 2012). Independent community‐level studies also support this possibility. Across the PETM, only minor increases in beta diversity occurred despite the immigration of new taxa, while some endemic fauna including phenacodontids expanded their geographic ranges (Darroch et al. 2014). Similarly, Fraser and Lyons (2020) found little change in overall community structure, body mass dispersion, or richness, suggesting that immigration did not substantially increase extinction or extirpation rates. Whittingham et al. (2024) likewise reported evidence of reduced environmental filtering during and after the PETM, with little evidence that competitive interactions strongly influenced community assembly. Collectively, these studies are consistent with the hypothesis that unsaturated niches promoted functional stability across the PETM. However, our DMTA results neither demonstrate nor refute this hypothesis; rather, they indicate that sympatric taxa consumed foods with broadly similar mechanical properties, a pattern compatible with but not unique to niche unsaturation.

An alternative explanation is microhabitat partitioning. Stable isotope analysis of herbivores from the early Eocene found that perissodactyls and phenacodontids exhibited lower δ^13^C and δ^18^O than artiodactyls (Secord et al. 2008), suggesting differences in microhabitat use (canopy coverage or drainage) or consumption of plants grown within different levels of the canopy. Because DMTA characterizes the mechanical properties of foods rather than the environments in which those foods were consumed, similar microwear textures could arise even if taxa foraged in different habitats. Consequently, our results neither support nor refute microhabitat partitioning. Integrating stable isotope analyzes with DMTA on the same specimens would provide a powerful means of testing whether taxa with similar dental microwear occupied different microhabitats or specialized on plants grown in specific levels of the canopy.

Neutral theory provides another possible explanation for the observed dietary overlap. Under this framework, multiple species may coexist despite similar resource use if demographic processes and low extinction rates reduce competitive exclusion (Bell 2000; Hubbell 2001). Notably, extinction rates in the Bighorn Basin during the PETM seemed to have been rather low, despite immigration of potential competitive species (Fraser and Lyons 2020; Gingerich 2003). An observed consequence of this is self‐organized similarity or clumped coexistence, where similar niches are occupied by multiple species and theoretically available niche spaces are left vacant (Codron et al. 2023; Scheffer and Van Nes 2006). The extensive overlap in dental microwear recovered here is compatible with this hypothesis, but these data do not directly evaluate neutral processes or community assembly. Consequently, neutral theory should be regarded as one ecological interpretation rather than a conclusion supported directly by the present analyzes.

Finally, it is also possible that differences in masticatory function and efficiency may be responsible for masking dietary diversity in dental microwear. Primary differences in dentognathic morphology suggest that mastication in phenacodontids was mostly compressive between the upper and lower dentition, with less translative chewing motions (Rensberger 1986). While phenacodontids like *Ectocion* developed more prominent ridges specialized for vertical shear, more “typical” phenacodontid molars possess low, obtuse cusps with only minor ridge development (Radinsky 1966). Because dental microwear reflects mastication patterns in addition to diet, it is possible that “archaic” chewing patterns do not record diet in the same way that more “modern” chewing patterns do (Charles et al. 2006; Teaford and Byrd 1989).

Collectively, our analyzes demonstrate that sympatric early ungulates likely consumed foods with similar mechanical properties despite differences in morphology and phylogenetic affinity. These results therefore constrain coexistence hypotheses by showing that strong dietary partitioning was limited. However, DMTA alone cannot determine whether coexistence was facilitated by niche unsaturation, spatial partitioning of similar resources, temporal differences in foraging, or neutral ecological processes. Resolving these alternatives will require integrating DMTA with complementary proxies, particularly stable isotope analyzes together with additional paleoenvironmental and community ecological data.

In general, anisotropy, complexity, scale of maximum complexity, and heterogeneity of complexity remained unchanged across all three lineages from the Clarkforkian through the PETM and into the early Wasatchian. In contrast, textural fill volume increased significantly only in *Ectocion* (Table 5, Figure 8). Textural fill volume is often strongly correlated with complexity, suggestive of brittle food consumption (Desantis et al. 2017; Scott 2012). This pattern of increasing textural fill volume without increasing complexity is perplexing. Therefore, any proposed explanation must account for an increase in textural fill volume while simultaneously explaining the absence of corresponding changes in the other DMTA variables.

One possible explanation suggests that dwarfing during the PETM altered the relative size of ingested particles with respect to tooth size, increasing textural fill volume even if foods remained broadly similar. Greater textural fill volume is suggestive of larger and/or deeper features and may be caused by the consumption of larger, brittle objects (Desantis et al. 2017). However, this hypothesis is weakened by the absence of comparable increases in *Copecion* and *Sifrhippus*, both of which experienced body‐size reductions of similar magnitude (Gingerich 2003; Secord et al. 2012). If body size alone drove the observed pattern, a more consistent response across lineages would be expected. Instead, the restriction of this shift to *Ectocion* suggests that lineage‐specific ecological or behavioral factors were also involved.

Alternatively, increased phytolith consumption represents a second plausible explanation because phytolith abundance has been found to be positively correlated with textural fill volume (Merceron et al. 2016). As microscopic amorphous silica‐based structures, phytoliths are secreted by plants intercellularly within the roots, stems, leaves, fruits, and flower parts (Nawaz et al. 2019), likely serving as responses to water and mineral stresses, defenses against pathogens and herbivores, and mechanical support for plants (Xu et al. 2023). However, Merceron et al. (2016) also reported a positive relationship between phytolith abundance and anisotropy, a pattern not observed in *Ectocion*. The absence of a significant increase in anisotropy therefore suggests that increased phytolith consumption alone is unlikely to fully explain the observed increase in textural fill volume.

Nevertheless, two phytolith‐related scenarios remain possible. First, increased aridity during the PETM may have promoted greater phytolith production within plants (Meunier et al. 2017; Xu et al. 2025) resulting in herbivores consuming broadly similar foods that contained higher concentrations of silica bodies. This explanation is weakened by the lack of a comparable increase in textural fill volume in *Copecion*, despite both genera inhabiting the same environment. Although the limited Clarkforkian sample size of *C. brachypternus* may have reduced statistical power, the available evidence does not support a basin‐wide increase in phytolith production as the sole driver of the observed pattern.

A second possibility is that *E. parvus* _PETM_ selectively consumed plant tissues with relatively high phytolith concentrations. Phytoliths occur throughout most plant organs but are typically most abundant in leaves and stems (Cormier and Armstrong 2025). They are also produced by numerous plant families that became more common in the Bighorn Basin during the PETM, including Annonaceae, Arecaceae, Fabaceae, Salicaceae, and Sapindaceae (Huisman et al. 2018; Mercader et al. 2009; Wing and Currano 2013). Although less common, phytoliths are also present within the pericarps of some fruits and seeds (Piperno 1989; Piperno et al. 2002). Selective consumption of these plant taxa or tissues could potentially elevate textural fill volume without requiring a basin‐wide increase in phytolith production.

Even this explanation is constrained by the absence of increased anisotropy, a measure that is also correlated with phytolith concentration (Merceron et al. 2016). One possibility is that *E. parvus* _PETM_ preferentially consumed phytolith‐bearing plant tissues that were not mechanically tough, such as pericarpal tissues of fruits and seeds. Alternatively, if *E. osbornianus*_Cf_ consumed mechanically similar plants containing few phytoliths, such as Arecaceae or Fabaceae (Ge et al. 2020), this could result in greater textural fill volume while producing only limited changes in the remaining DMTA variables. Although these scenarios remain plausible, the lack of a corresponding anisotropy increase suggests that phytolith enrichment alone is unlikely to provide a complete explanation for the observed microwear pattern.

Increased contact with exogenous grit resulting from greater aridity during the PETM also remains a viable explanation for the observed increase in textural fill volume in *E. parvus* _PETM_. The role of dust and grit on the formation of dental microwear remains fiercely debated, with studies reaching opposing conclusions (Ackermans et al. 2020; Adams et al. 2020; Merceron et al. 2016). Nevertheless, both in vitro and in vivo experiments demonstrate that exogenous particles can generate and modify dental microwear (Hua et al. 2015; Schulz‐Kornas et al. 2020; Teaford et al. 2021). Moreover, increasing evidence suggests that grit and diet interact to form dental microwear, with the effects of exogenous abrasives dependent on food type rather than being able to completely mask dietary signals (Hua et al. 2020; Merceron et al. 2016).

The greater aridity of the PETM likely increased the abundance of dust and grit within the Bighorn Basin (Achakulwisut et al. 2019), potentially increasing incidental ingestion of abrasive particles during feeding. Studies have shown that overall microwear volume is more strongly influenced by abrasive size than quantity (Ackermans et al. 2020; Winkler, Tütken, et al. 2020). Consequently, the observed increase in textural fill volume could reflect greater exposure to larger exogenous abrasives without necessarily producing corresponding changes in anisotropy or complexity, making this mechanism broadly consistent with the DMTA pattern recovered for *Ectocion*. However, because the independent effects of exogenous grit on DMTA variables remain incompletely resolved, this hypothesis cannot presently be distinguished from lineage‐specific dietary shifts. Experimental studies examining the effects of grit size and abundance on dental microwear textures are therefore needed to better interpret the increase in textural fill volume across the PETM.

Overall, the observed increase in textural fill volume without accompanying changes in other dental microwear variables argues against a simple increase in brittle food consumption and provides only limited support for a basin‐wide increase in phytolith abundance. Instead, the available evidence is more consistent with either lineage‐specific shifts in resource use or increased exposure to coarse exogenous abrasives during the PETM. Distinguishing between these alternatives will require experimental studies investigating the independent effects of phytoliths, grit, and relative ingesta size on dental microwear formation.

Beyond differences in textural fill volume within *Ectocion*, failure to recover significant changes in dental microwear across the PETM provides insight into dietary responses during this period of intense climatic warming and drying, as well as the immigration of new taxa into the Bighorn Basin. However, this interpretation of null results must consider limitations imposed by fossil sample availability. Empirical power analyzes indicate that several comparisons lacked sufficient specimens to reliably detect moderate effects, particularly within taxa with small sample sizes. Interpretation of the absence of significant dietary change in *Copecion* is particularly constrained by small and uneven sample sizes across biozones. Although the PETM and Wasatchian samples include 12 and 14 specimens, respectively, the Clarkforkian sample consists of only four specimens, limiting the ability to detect subtle temporal dietary shifts. Similarly, detecting a moderate temporal dietary shift in *Sifrhippus* (VDA ≥ 0.64) would require substantially more specimens than currently available. Additional specimens, particularly of Clarkforkian *C. brachypternus*, are necessary to determine whether more subtle dietary changes occurred. Nevertheless, the repeated absence of significant differences across these three independent lineages provides no evidence for widespread shifts in dietary mechanics as a ubiquitous response to PETM environmental change.

Phylogenetic dwarfing among these three lineages during the PETM has been variably explained by increasing temperature or decreased dietary protein consumption (Gingerich 2006; Secord et al. 2012). Intense floral turnover and reductions in primary productivity as a cause for body mass reduction would likely result in dietary change that may be reflected in dental microwear. Regardless of cause, it still may be expected that dental microwear changed during the PETM. Plants grown under conditions of gradual dehydration that simulate the aridity of the PETM show stems and leaves that exhibit greater bending stiffness and fracture toughness, respectively (Gerolamo et al. 2022). It would be expected that the dental microwear of herbivores would reflect these plant biomechanical changes, regardless of body mass trends across the PETM. Considering these results of limited dental microwear change, alternative hypotheses related to selective foraging ecology and the degree to which plant biomechanical properties adjusted to the PETM require further investigation to more completely understand the diets of these ancient herbivores.

Evaluating how plants and animals responded to the biotic and abiotic disruptions of the PETM, including dramatic warming and drying, as well as the immigration of new flora and fauna provides important context for understanding ecological responses to environmental change, particularly among modern ecosystems. However, the PETM should not be viewed as a direct analog for future climate change due to the substantially faster rate of current and projected climatic warming and the anthropogenic impacts faced by contemporary species being absent in deep time ecosystems. Thus, the limited dietary responses observed in the taxa examined here should not be interpreted as evidence that modern species and ecosystems will remain similarly stable under future climate change. Instead, the PETM provides a deep time perspective for understanding how ecological interactions and dietary strategies may respond to environmental change, while highlighting the importance of considering the broader context of climate change when anticipating future ecological responses. Ultimately, understanding how dietary ecology changed across the PETM can therefore provide valuable context for anticipating how modern species and ecosystems may respond to ongoing environmental change.

## Author Contributions

**Andrew Schwartz:** conceptualization (lead), data curation (lead), formal analysis (lead), funding acquisition (equal), investigation (lead), methodology (equal), project administration (lead), resources (equal), software (equal), validation (lead), visualization (lead), writing – original draft (lead), writing – review and editing (lead). **Larisa R. G. DeSantis:** data curation (supporting), funding acquisition (equal), investigation (supporting), methodology (supporting), project administration (equal), resources (equal), software (lead), supervision (lead). **Rob S. Scott:** methodology (supporting), project administration (equal), supervision (equal), validation (equal), writing – review and editing (supporting).

## Funding

This work was made possible by the Albert Fellows Award funded by the Center for Human Evolutionary Studies (CHES) at Rutgers University. This work was performed on equipment funded by the National Science Foundation (grant ID 1053839 to DeSantis).

## Conflicts of Interest

The authors declare no conflicts of interest.

## Data Availability

The data generated by this study as well as the script for these analyzes are available at Dryad (https://doi.org/10.5061/dryad.1c59zw4bc).
